# Increasing multi-hazard climate risk and financial and health impacts on northern homeowners

**DOI:** 10.1007/s13280-023-01951-z

**Published:** 2023-11-13

**Authors:** Tobias Schwoerer, Jennifer I. Schmidt, Matthew Berman, Peter Bieniek, Louise M. Farquharson, Dmitry Nicolsky, James Powell, Rachel Roberts, Rick Thoman, Robert Ziel

**Affiliations:** 1 International Arctic Research Center, University of Alaska Fairbanks, PO Box 75734, Fairbanks, AK 99775-7340 USA; 2 Institute of Social and Economic Research, University of Alaska Anchorage, 3211 Providence Dr., Anchorage, AK 99509 USA; 3 Geophysical Institute, University of Alaska Fairbanks, PO Box 757320, Fairbanks, AK 99775-7340 USA; 4 Alaska Coastal Rainforest Center, University of Alaska Southeast, 11066 Auke Lake Way, Juneau, AK 99801 USA

**Keywords:** Arctic, Climate risk, Hazard mitigation, Permafrost, Rain-in-winter, Wildfire

## Abstract

Currently, more than half of the world’s human population lives in urban areas, which are increasingly affected by climate hazards. Little is known about how multi-hazard environments affect people, especially those living in urban areas in northern latitudes. This study surveyed homeowners in Anchorage and Fairbanks, USA, Alaska’s largest urban centers, to measure individual risk perceptions, mitigation response, and damages related to wildfire, surface ice hazards, and permafrost thaw. Up to one third of residents reported being affected by all three hazards, with surface ice hazards being the most widely distributed, related to an estimated $25 million in annual damages. Behavioral risk response, policy recommendations for rapidly changing urban environments, and the challenges to local governments in mitigation efforts are discussed.

**Supplementary Information:**

The online version contains supplementary material available at 10.1007/s13280-023-01951-z.

## Introduction

Over coming decades and centuries, climate change is predicted to intensify weather-related impacts on urban infrastructure and urban populations across the globe (IPCC 2022). While most research has focused on cities in lower latitudes and investigated single hazards, there is a need to understand more fully how multiple climate change-related hazards affect urban residents, specifically in northern latitudes (Binita et al. 2021). These residents respond to increasing climate risk stemming from multi-hazard urban environments driven by Arctic warming at almost four times the global average (Rantanen et al. 2022). But little is known about the impacts of various climate-related hazards and their economic effects on urban residents in the North (Berman and Schmidt 2018; Markon et al. 2018). Recent research has focused on health impacts (Carter et al. 2016; Zhang et al. 2020; Ebi et al. 2021), and adaptive capacity of urban residents to respond (Glaas et al. 2015; Neset et al. 2016; Ballantyne et al. 2018).

While some climate-related hazards in urban environments parallel those of lower latitudes, such as flooding (Nie et al. 2009), high winds (Steenbergen et al. 2012), more frequent wildfires (Calef et al. 2015), and heat (Esau et al. 2021), other hazards are unique to the North such as permafrost thaw threatening built infrastructure (Hjort et al. 2018) and rain-in-winter events (Cohen et al. 2015). Rain-in-winter or freeze thaw cycles, sometimes without precipitation, lead to surface ice hazards that can cause bodily injury and property damage (Black and Mote 2015). Compared to lower latitudes, research on the impacts of climate-change-related hazards on urban residents in northern latitudes has received little attention (Binita et al. 2021). Studies addressing hazards in Alaska focused on public investments needed for mitigation (Melvin et al. 2017; Streletskiy et al. 2019) or health risk (Haney 2020; Woo et al. 2020), but did not consider economic effects for private homeowners.

The research focused on two northern communities in Alaska, USA, assessing three environmental hazards: wildfire, surface ice, and permafrost thaw with the objectives to quantify private climate risk and mitigation investments, elicit risk perceptions, and quantify hazard impact on local livelihoods. Together with local risk management personnel, a homeowner survey was co-designed. Below, we describe the study communities, methods for analysis followed by survey response and results. The discussion compares results from the two cities and presents policy recommendations for mitigation planning in northern cities specifically, and generally in multi-hazard environments.

### Study area

The Municipality of Anchorage and the Fairbanks North Star Borough, including the cities of Fairbanks and North Pole, belong to Alaska’s largest urban areas with 2020 U.S. Census populations of 291 247 and 95 665 residents and median household incomes of $88 871 and $78 321, respectively (U.S. Census 2021). The owner-occupied housing rate was 63% in Anchorage and 59% in Fairbanks (U.S. Census 2021).

**Fig. 1:**
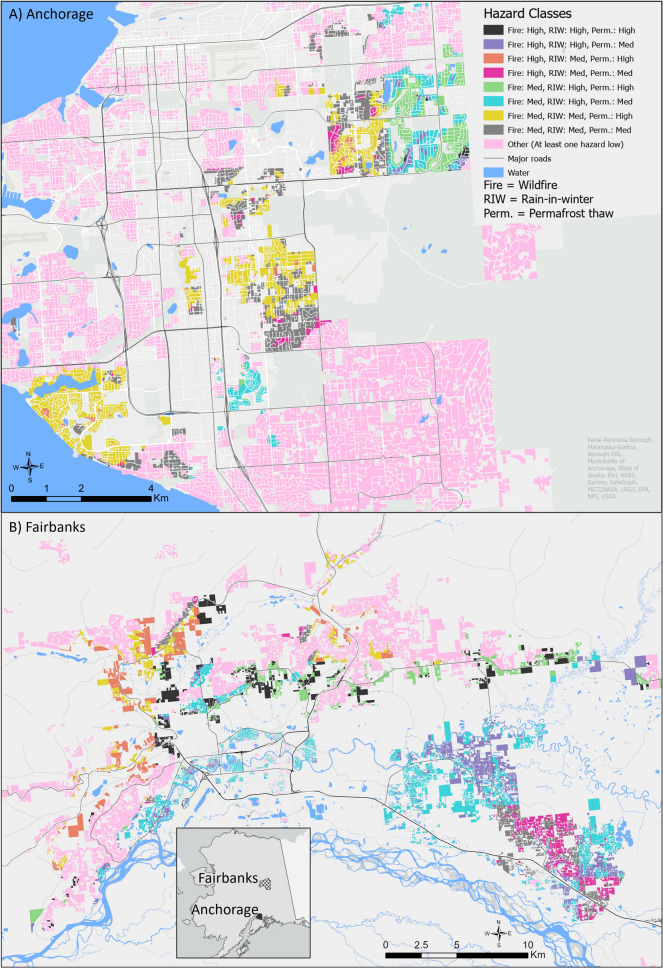
Map showing the results of a hazard assessment for wildfire, rain-in-winter, and permafrost in Anchorage (**A**) and Fairbanks (**B**), Alaska, USA. Areas are assigned low, medium, or high hazard levels. The colored areas show multi-hazard areas with unique combinations of hazard levels across the three hazards. For detailed hazard assessment maps, see Supplementary Information 1

Anchorage is in Southcentral Alaska about 400 km south of Fairbanks in the Interior of Alaska (Fig. 1). Anchorage has a subarctic climate with marine influences that results in a moderate climate. The mean temperature (mean precipitation) between 1993 and 2023 for January was –8.3 degrees C (19 mm) and for July equaled 15.4 degrees C (46 mm) (NOAA 2023). Fairbanks is the coldest large city in the U.S. and has a humid continental climate with long very cold winters and short warm summers. The mean temperature (precipitation) between 1993 and 2023 for January was − 22.4 °C (15.5 mm) and for July equaled 17.1 °C (57.4 mm) (NOAA 2023).

Both Anchorage and Fairbanks are in the North American Boreal Forest biome. Urban development in both cities has expanded into forested lands susceptible to wildfire and resulted in an increase of the wild urban interface (Grabinski and McFarland 2020). Both Anchorage and Fairbanks participate in the *Firewise* program, a public education program that state and local governments participate in (NFPA 2023). It is designed to help protect private property threatened by wildfire. The communities partially reimburse homeowners for their mitigation expenses suggested by the program, develop community wildfire protection plans, and coordinate with state and federal fire suppression efforts (Grabinski and McFarland 2020).

To reduce risk from wildfire, homeowners can undertake various mitigation activities on the land surrounding a home and on the home itself. Radiant heat from burning vegetation surrounding the structure can ignite the structure (Molina et al. 2017). Consequently, creating defensible space by clearing dead trees and shrubs and by pruning and thinning coniferous trees close to the home is an important mitigation strategy (NFPA 2023). In addition, selecting non-flammable home building materials, especially for siding, decking, and roofing can also protect a structure against wildfire. Lastly, homeowners reduce wildfire hazard by cleaning gutters, using vent screens, and promoting fire-resistant vegetation (NFPA 2023).

In Alaska, surface ice hazards that form through rain-in-winter events or freeze thaw cycles occur frequently over southwestern and southern coastal regions, including Anchorage (Bieniek et al. 2018), but are currently more infrequent in interior regions such as Fairbanks (Pan et al. 2018; Serreze et al. 2021). These events can have long-lasting impacts because of the prevailing sub-freezing temperatures and minimal solar heating in mid-winter. As temperatures in northern latitudes increase, the extent and frequency of ice hazards are likely to increase in both communities (Pan et al. 2018).

Permafrost is widely distributed in areas around Fairbanks with high to moderate ground ice content (Fig. 1) (Péwé and Bell 1975; Pewe and Reger 1989). Ice-rich permafrost is extremely susceptible to subsidence and the formation of hummocky terrain termed thermokarst when it thaws (Jorgenson et al. 2006). Surface disturbances such as land clearing and snow piling that influence the ground thermal regime promote thermokarst formation (O’Neill and Burn 2017). Wildfire may cause some types of permafrost to deteriorate, illustrating that the three hazards we focus on can have compounding effects, requiring carefully planned mitigation actions in zones with overlapping wildfire and permafrost hazards (Shur and Jorgenson 2007). Permafrost is not widespread in Anchorage, as the mean annual air temperature is above freezing (Kanevskiy et al. 2013). Comparing permafrost distributions between Anchorage and Fairbanks, the “high” category in Anchorage is roughly equivalent to the “low” in Fairbanks (Fig. 1). However, in Anchorage, patches of permafrost occur sometimes containing relic glacial ice that, when thawed would result in surface subsidence (Fig. 1) (Shur and Jorgenson 2007; Kanevskiy et al. 2013).

## Materials and methods

The research conducted a homeowner survey to investigate the impacts of multiple climate-related hazards on private homeowners, measured risk perceptions, analyzed mitigation response, and estimated related costs associated with mitigation and property damage or bodily injury. A geospatial hazard assessment informed the survey’s sampling design. The conceptual and theoretical framework for the study lies in social-ecological systems inquiry, specifically in exploring the relationships between society and the environment (Folke et al. 2016). Other relevant theoretical work relates to human behavior, choice, and values under risk and uncertainty (Tversky and Kahneman 1974). Below, we describe the sample, survey design, and data analysis.

### Sample and survey design

Two stratified random samples were drawn, each from property tax bases maintained by local governments, one containing 2000 of 82 148 properties in Anchorage, and the other containing 2000 of 18 074 properties in the Fairbanks area including the cities of Fairbanks and North Pole (Supplementary Information 1, Tables S1 and S2). The research relied on hazard mapping to assign one of three hazard levels to each of the three hazards: wildfire (Schmidt et al. 2022), surface ice (Berman et al. 2022), and permafrost (Nicolsky et al. 2021). The sampling used proportional probability to size without replacement (Chromy 2011) and drew proportionally across 27 possible strata (Supplementary Information 1).

Together with risk management personnel from local emergency management organizations and private property owners, we co-designed a homeowner survey to better understand the three hazards and local hazard response (Norström et al. 2020). The research team held four focus groups in Anchorage and two in Fairbanks with 27 participants and interviewed 37 local experts and risk management personnel (Wilkinson 1998; Powell et al. 2023). The study followed Dillman’s tailored design method (Dillman et al. 2014), iteratively revising versions of the survey instrument, and targeting a high level of relevancy to both respondents and risk response personnel (Djenontin and Meadow 2018). Finally, we conducted a pretest with 50 randomly assigned homeowners that achieved four responses which were used to improve survey flow, clarity, and to eliminate ambiguity.

The survey started with three sections specific to each hazard, asking questions about personal experiences, impacts, and hazard mitigation (Supplementary Information 1). Each hazard-specific section ended with an exercise measuring the respondent’s perceived risk (Slovic 2016) using a 10-point semantic differential scale question that asked respondents to rate their subjective risk associated with the hazard (Weber and Borcherding 1993; Martin et al. 2012). We refer to the latter as subjective risk ratings. The survey ended with questions about insurance coverage, hazard mitigation costs, and demographics. Administration of the survey occurred between February and March 2022 through an online survey platform (Qualtrics 2021). Each of the 4000 homeowners received a letter of invitation by mail containing an individualized URL to the online survey and a $2 bill as a token of appreciation. After the initial mailing, we sent a reminder post card, and a final reminder letter, each 2 weeks apart. The study was approved by the University of Alaska Fairbanks Institutional Review Board (IRB) under protocol 1754769 that ensured human subjects protection and research participants were made aware that their participation in the study was voluntary, that they could leave the project at any time, and that their names and identities would not be shared.

### Data analysis

Iterative proportional fitting (raking) by household income was used for estimating proportions and means and to adjust for nonresponse while accounting for the stratified survey design (Kalton 1986; DeBell and Krosnick 2009). Income had high response (95%) and could be compared to Census data (Pike 2007; U.S. Census 2021). For the survey analysis, we used the *svydesign* and *rake* functions in R’s *survey* package (Supplementary Information 2) (Lumley 2010; R Core Team 2022). For the population margins associated with the weighting variable in the *rake* function, we generated frequency tables for income categories as defined in the survey instrument (U.S. Census 2022).

For multiple choice questions with open-ended text entries, we applied qualitative coding analysis assigning responses to existing or creating additional categories (Auerbach and Silverstein 2003) (Supplementary Information 2). We removed the following outliers, one stating $25 000 for annual average fire mitigation costs and one reporting $250 000 in permafrost related mitigation. We also removed nine outliers that showed person-hours for wildfire mitigation exceeding 1000 person-hours a year.

## Results

Below, we present survey response and sample characteristics followed by a section on multi-hazards and income relationships. For each hazard separately, we then present impacts, perceived risk, and estimated mitigation response and costs. We provide additional survey results in Supplementary Information 1. The anonymized data are archived and publicly available (Schwoerer 2023).

### Survey response and sample characteristics

Of the 4000 total mailings to residents in Anchorage and Fairbanks, 413 were undeliverable. A total of 379 Fairbanks and 320 Anchorage homeowners participated in the survey for an overall response rate of 19.5% (*n* = 722) (Supplementary Information 1 Table S3). Respondents from Fairbanks on average lived 2.5 years longer at their residences than respondents from Anchorage (*n* = 420). The largest proportion of respondents were between 61 and 70 years old (23% in Anchorage and 25% in Fairbanks), followed by the 41-to-50 (22% in Anchorage, 20% in Fairbanks) and 51-to-60-year age groups (21% in Anchorage, 22% in Fairbanks) (*n* = 710).

The survey had good coverage across strata (Supplementary Information 1 Table S4). Of the 16 existing hazard zones in Anchorage, only one zone including four contacts did not respond. Out of the 20 mapped hazard zones in Fairbanks, only two zones containing five contacts did not respond. In Anchorage, the survey had disproportionally higher response rates in the high wildfire and high surface ice hazard zones and fewer responses in the high permafrost hazard zone (Supplementary Information 1 Table S4). In Fairbanks, response in the high wildfire zone was also disproportionally higher, similarly to the high permafrost zone.

### Income and hazards

**Fig. 2:**
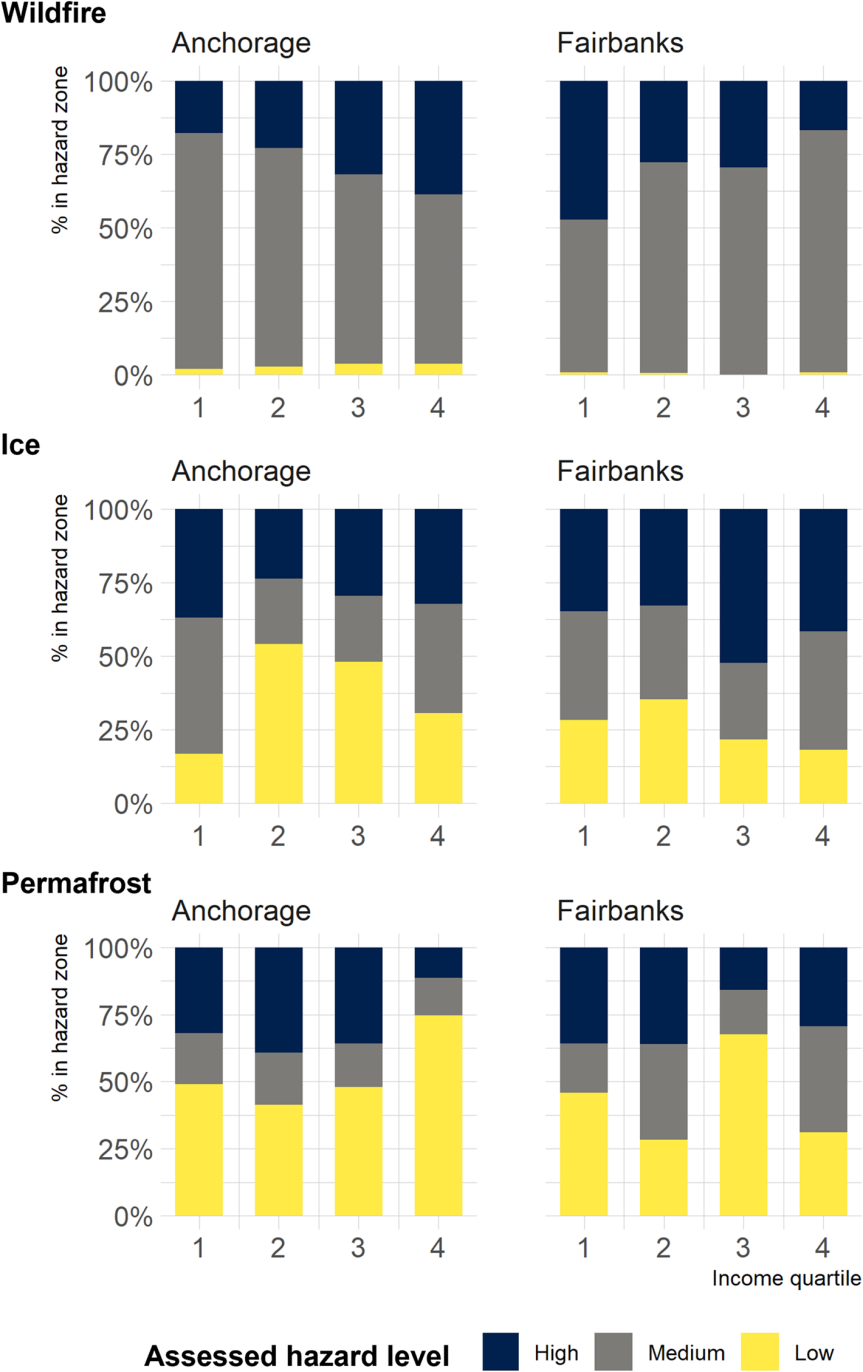
Percentage of homeowners residing in each hazard zone by reported income quartile, *n* = 589; showing lower-income homeowners in Fairbanks are more likely to reside in the high wildfire hazard zone, opposite to Anchorage. Lower-income homeowners are also more likely to reside in the high permafrost hazard zone, especially in Fairbanks

In Fig. 2, we illustrate the percentages of homeowners residing in each hazard zone given income. In Anchorage, a higher proportion of higher-income homeowners in the 3rd and 4th quartile (35%) resided in the high wildfire hazard zone compared to 19% of lower-income homeowners in the 1st and 2nd quartile. In Fairbanks, this effect is the opposite, with 40% of lower-income homeowners residing in the high wildfire hazard zone compared to 21% of higher-income homeowners. Surface ice hazards had a similar, opposing correlation, between the two cities. In Anchorage, 31% of higher-income homeowners resided in the high surface ice hazard zone compared to 34% of lower-income homeowners. In Fairbanks, 45% of higher-income homeowners resided in the high surface ice hazard zone compared to 34% of lower-income homeowners. Permafrost tends to affect lower-income homeowners more in both cities with the effect more pronounced in Fairbanks where 36% of lower-income homeowners resided in the high permafrost hazard zone in contrast to 25% of higher-income homeowners. In Anchorage, these percentages were 33% and 27%, respectively. Supplementary Information 1 shows the number of homeowners by income quartile and hazard level.

### Multi-hazards

The estimated number of homeowners affected by at least one of the three hazards varied by city, with Fairbanks homeowners being relatively more affected by multi-hazards (Table 1). Almost twice as many homeowners were affected by all three hazards in Fairbanks (6264) compared to Anchorage (3282) (Fig. 1), with larger proportional differences (35% vs. 4%) due to the relatively larger Anchorage population. In both cities, the combination of impacts from wildfire and surface ice was most frequent. Most homeowners in Anchorage (58%) were solely affected by surface ice hazards alone, while in Fairbanks, most homeowners (71%) were affected by at least two hazards (Table 1).

**Table 1:** Estimated number of homeowners affected by multiple hazards, based on reported impacts, *n* = 683

Multi-hazards	Anchorage		Fairbanks	
	Est. count	%	Est. count	%
Affected by all three hazards	3282	4	6264	35
Affected by two hazards	20 036	25	6419	36
Wildfire & surface ice	11 154	14	3530	20
Permafrost & surface ice	7960	10	2349	13
Permafrost & wildfire	922	1	540	3
Affected by only one hazard	49 239	59	4809	26
Surface Ice	47 867	58	4593	25
Wildfire	1153	1	75	< 1
Permafrost	219	< 1	141	1
Not affected by either hazard	9591	12	583	3
Parcels, *n*	82 148		18 074	

### Wildfire

The impacts of wildfire on people’s livelihoods were more widespread in Fairbanks compared to Anchorage and included having to stay indoors (14% Anchorage, 43% Fairbanks), reducing outdoor activities (13%, 48%), facing interruptions in electricity and Internet services (1%, 5%), and inconvenience of evacuations and access restrictions (5%, 16%) (Supplementary Information 1 Table S5). More severe impacts included loss of property, temporary or extended evacuations, and respiratory problems.

The subjective risk ratings on a ten-point scale were on average 1.7 points lower in Anchorage compared to Fairbanks (Table 2). Properties located in the high wildfire hazard zone in Fairbanks were three times as likely to report being affected by wildfire than properties in the respective zone in Anchorage. In addition, a higher percentage (56%) of homeowners in Fairbanks believed that the wildfire hazard worsened over the past decade, compared to Anchorage (33%).

**Table 2:** Homeowners’ subjective risk rating, mitigation investment and effort, and perceived hazard trend over the past 10 years for wildfire

	Subjective risk rating^a^ Mean (SE)	*n*	Affected homeowners % (SE)	*n*	5-year mitigation cost^b)^ Mean (SE)	*n*	Mitigation effort in person- hours/yr Mean (SE)	*n*	Firewise participation % (SE)	*n*	Homeowners stating worsening hazard % (SE)	*n*	Homeowners supporting elect. utilities’ power shutoff % (SE)	*n*
**Anchorage**	3.1 (0.2)	290	20% (4%)	61	$1706 (608)	10	40 (6)	228	34% (9%)	59	33% (4%)	294	59% (5%)	227
High	4.2 (0.6)		23% (7%)		$1051 (652)		54 (19)		57% (20%)		57% (9%)		61% (9%)	
Medium	3.0 (0.2)		22% (6%)		$1863 (735)		41 (5)		n/a		28% (5%)		52% (5%)	
Low	1.7 (0.2)		0% (0%)		n/a		11 (4)		24% (7%)		26% (24%)		95% (4%)	
**Fairbanks**	4.8 (0.7)	359	58% (9%)	141	$510 (32)	23	54 (5)	281	28% (20%)	83	56% (16%)	361	79% (10%)	275
High	5.5 (1.1)		71% (9%)		$500 (7)		43 (8)		10% (9%)		70% (23%)		89% (8%)	
Medium	4.3 (0.3)		47% (7%)		$577 (190)		66 (11)		70% (12%)		44% (7%)		72% (8%)	
Low	2.8 (0.8)		10% (38%)		n/a		44 (27)		64% (75%)		9% (38%)		12% (48%)	

^a^A rating scale between 0 and 10 was used with 10 being the highest risk

^b^On average, respondents reported paying out-of-pocket 69% (SE 16%) in Anchorage and 98% (SE 2%) in Fairbanks with the remainder covered by insurance. An outlier of $25 000 was removed for calculating the mean. The estimated mitigation expenses for wildfire in both Fairbanks and Anchorage were based on a very small sample but are within the range of willingness to pay estimates for Alaska residents (Molina et al. 2021)

In Fairbanks, a much larger percentage of homeowners had homeowners’ insurance that covered wildfire with almost two thirds (63%) of homeowners insured against wildfire as opposed to Anchorage where less than a third (29%) had this coverage. Few homeowners (2%) reported that insurance companies required them to prepare the property for wildfire. A similar percentage thought wildfire coverage was too expensive or not worth the cost. Two percent of homeowners in Fairbanks and 1% in Anchorage stated not to be eligible for wildfire coverage.

**Fig. 3:**
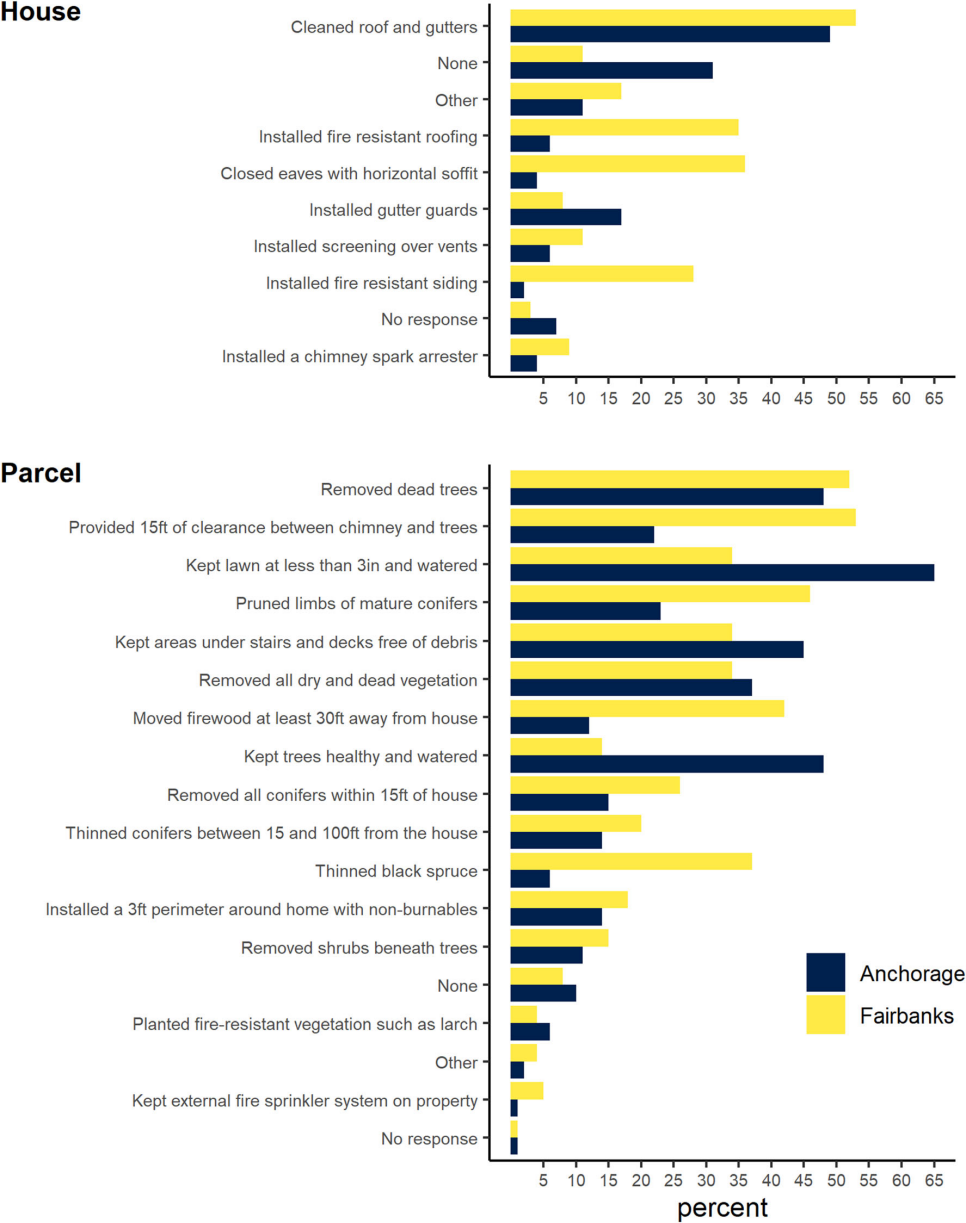
Estimated percentage of homeowners reporting to have completed wildfire mitigation activities on their house and land parcel over the past 5 years; Anchorage *n* = 316, Fairbanks *n* = 375

A larger percentage of homeowners in Fairbanks completed at least some wildfire mitigation on their houses in the past 5 years with 89% in Fairbanks compared to 69% in Anchorage (Fig. 3). About a third of Fairbanks homeowners installed fire-resistant siding, fire-resistant roofing, and closed eaves with horizontal soffits to reduce the risk of wildfire ignition. A much smaller percentage took these steps in Anchorage. Fairbanks homeowners also showed higher mitigation levels on their land parcels (Fig. 3). For example, almost half (46%) of homeowners in Fairbanks pruned limbs from mature conifers and 42% moved firewood at least 30 ft away from the house, compared to 23% and 12% of Anchorage homeowners.

Total estimated wildfire mitigation expenses between 2017 and 2021 amounted to $2.1 million (SE: 1.0 million) in Anchorage and $2.6 million (SE: 1.8 million) in Fairbanks. The total number of person-hours per year spent on mitigating wildfire hazards was estimated at 2.3 million (SE: 379 048) person-hours annually for Anchorage and 786 655 (SE: 119 128) for Fairbanks. On average, Fairbanks property owners spent 30% more-time mitigating wildfire hazards compared to Anchorage homeowners, 54 vs. 40 person-hours per year (Table 2).

Most homeowners in Anchorage (81%) and Fairbanks (96%) believed that private landowners have a responsibility to prepare their home and surrounding landscape for wildfire (Supplementary Information 1 Table S8). Thirty percent in Anchorage and 44% in Fairbanks believed that landowners should participate in wildfire protection programs such as *Firewise*. We estimate that 3766 homeowners in Anchorage and 2051 in Fairbanks participated in *Firewise*, corresponding to 34% of private property owners in Anchorage and 28% in Fairbanks (Table 2). While *Firewise* participation in the high wildfire hazard zone in Anchorage was higher compared to lower hazard zones, the opposite was true for Fairbanks. In Fairbanks, *Firewise* participation was much higher in the lower and medium hazard zones (70% and 64%) compared to the high hazard zone (10%).

Public support for proactive electricity shut offs during high fire danger, such as accompanied by high winds, was stronger in Fairbanks where 79% supported this policy compared to Anchorage, 59% (Table 2 and Supplementary Information 1 Table S6). Public perception of the government’s effectiveness and preparedness in responding to wildfires varied between the two cities with Fairbanks homeowners being more confident in government wildfire response (Supplementary Information 1 Table S7).

### Surface ice hazards

**Fig. 4:**
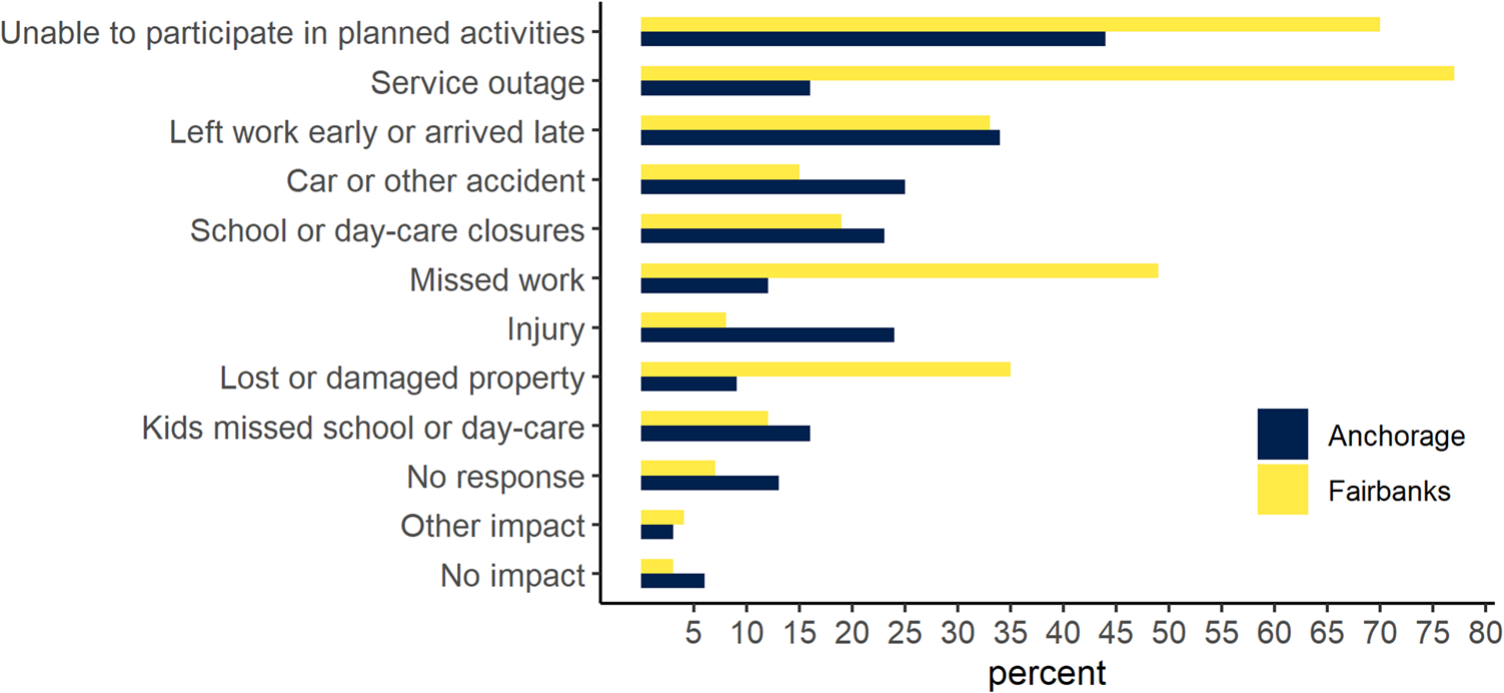
Estimated percentage of homeowners reporting to have been affected by surface ice hazards, Anchorage *n* = 278, Fairbanks *n* = 352

Most homeowners in both Anchorage and Fairbanks were affected by surface ice hazards; 85% in Anchorage and 93% in Fairbanks with varying impacts from missed work and service interruptions to more serious consequences related to accidents resulting in injury and lost or damaged property (Fig. 4, Table 3). In 2020 and 2021, more serious consequences were reported by a quarter of Anchorage residents and 15% in Fairbanks (Fig. 4). On average, homeowners’ mean cost for health care and damages related to surface ice hazards in the past 2 years were $899 in Anchorage compared to $471 in Fairbanks. The estimated number of homeowners who had ice-related property damage over the same period was 25 079 in Anchorage and 3902 in Fairbanks amounting to 43% and 26% of homeowners, respectively. Surface ice-related total damage and health care costs over the past 2 years were estimated to equal $47.8 million (SE 13 million) in Anchorage and $6.7 million (SE 3.3 million) in Fairbanks. Surface ice-related service outages were reported by 77% in Fairbanks and 16% in Anchorage with most outages in Fairbanks lasting between a day and a week and most in Anchorage lasting less than a day. Less than 3% reported outages lasting more than a week.

**Table 3:** Homeowner subjective risk ratings, estimated mitigation investments, estimated damage and health care costs, and perceived hazard trend over the past 10 years for surface ice

	Subjective risk rating^a^ Mean (SE)	*n*	Affected homeowners % (SE)	*n*	2-year mitigation cost^b^ Mean (SE)	*n*	2-year damage and health care cost^c^ Mean (SE)	*n*	Homeowners with damages, injuries in past 2 years % (SE)	*n*	Homeowners stating worsening hazard % (SE)	*n*
**Anchorage**	5.3 (0.3)	298	85% (1%)	275	$632 (89)	272	$899 (239)	184	43% (6%)	209	51% (6%)	289
High	5.3 (0.6)		85% (5%)		$692 (140)		$1 785 (770)		53% (12%)		47% (10%)	
Medium	5.7 (0.4)		91% (3%)		$564 (165)		$866 (304)		51% (10%)		44% (8%)	
Low	5.0 (0.4)		80% (10%)		$658 (169)		$364 (204)		28% (10%)		61% (9%)	
**Fairbanks**	6.2 (0.6)	362	93% (2%)	332	$781 (131)	314	$471 (242)	232	26% (8%)	263	86% (9%)	348
High	6.3 (0.9)		94% (4%)		$965 (170)		$591 (334)		42% (12%)		86% (16%)	
Medium	6.9 (0.5)		94% (4%)		$619 (51)		$316 (239)		9% (5%)		93% (4%)	
Low	4.4 (0.4)		88% (7%)		$871 (400)		$683 (503)		41% (18%)		72% (15%)	

^a^A rating scale between 0 and 10 was used with 10 being the highest risk

^b^On average, respondents reported paying out-of-pocket 75% (SE 7%) in Anchorage and 59% (SE 19%) in Fairbanks with the remainder covered by insurance

^c^Out-of-pocket 56% (SE 15%) in Anchorage and 40% (SE 24%) in Fairbanks. Average commuting times are 19 min in both cities (U.S. Census 2021)

Homeowners’ perceived risk as measured by the subjective risk rating exercise for surface ice hazards did not vary much across the assessed hazard areas in Anchorage and only varied between low and medium/high hazard areas in Fairbanks (Table 3). On the ten-point subjective risk-rating scale, Fairbanks homeowners rated surface ice hazards 0.9 points higher compared to Anchorage homeowners. The percentage of homeowners believing that the surface ice hazard has worsened in the past decade was higher in Fairbanks with 86% compared to Anchorage, 51%.

Reported actions to mitigate surface ice hazards were more prevalent in Anchorage than Fairbanks and ranged from spreading sand, gravel, and ice melt to removing hard packed snow and purchasing slip-resistant shoes (Supplementary Information 1 Table S9). Almost 80% of Anchorage homeowners reported having purchased slip-resistant shoes or ice cleats in contrast to 9% in Fairbanks. Only 4% of Fairbanks residents purchased studded snow tires in the past 2 years, whereas 50% purchased them in Anchorage. This result is consistent with most homeowners in Fairbanks (52%) using snow tires without studs compared to 23% in Anchorage (Supplementary Information 1 Table S10). Over the past 2 years, private homeowners spent an estimated $45.6 million (SE 6.4 million) on ice-hazard-related mitigation in Anchorage compared to $12.8 million (SE 2.3 million) in Fairbanks.

### Permafrost

**Fig. 5:**
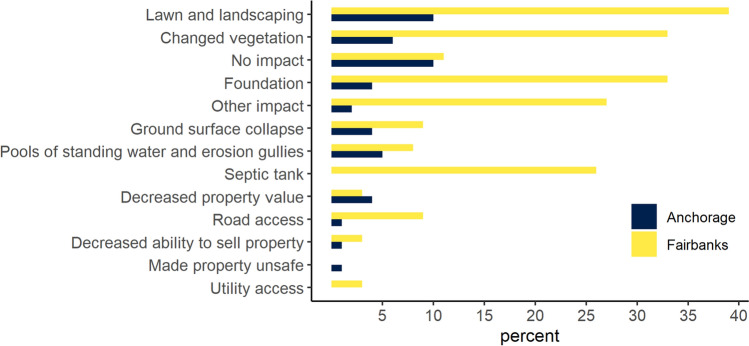
Estimated percentage of homeowners reporting to have been affected by permafrost thaw, Anchorage *n* = 73, Fairbanks *n* = 157. Note, homeowners in Anchorage with septic systems do not generally live in areas with permafrost hazard

Impacts from thawing permafrost were predominantly reported in Fairbanks, where 51% of homeowners were affected by permafrost thaw compared to 15% in Anchorage (Table 4). More than a third (39%) of Fairbanks homeowners observed changes to lawn and landscaping, 33% reported having issues with their foundation (4% in Anchorage), and 25% in Fairbanks mentioned issues with their septic system (Fig. 5). More than a quarter (27%) of Fairbanks homeowners reported to also have had other impacts, including fence posts jacking, porch and deck and other outdoor installments heaving, driveway heaving, power pole jacking, exposed water/waste-water utility lines, permafrost affecting garden and vegetable growth, spruce trees tipping over, unbalanced settling of house foundation, thaw affecting trees and hydrology, and driveway heaves and low spots. Less than 5% believed to be affected by decreasing property values due to permafrost thaw.

**Table 4:** Homeowner subjective risk ratings, estimated mitigation costs, and perceived hazard trend over the past 10 years for permafrost

	Subjective risk rating^a^ Mean (SE)	*n*	Affected homeowners % (SE)	*n*	Mitigation cost^b^ Mean (SE)	*n*	Stated worsening hazard % (SE)	*n*
**Anchorage**	1.9 (0.3)	194	15% (4%)	73	$3717 (1606)	23	31% (9%)	294
High	2.1 (0.3)		10% (4%)		$3783 (942)		15% (10%)	
Medium	2.9 (0.6)		29% (11%)		$7101 (942)		49% (23%)	
Low	1.4 (0.3)		11% (3%)		$718 (508)		31% (9%)	
**Fairbanks**	3.4 (0.7)	276	51% (13%)	157	$31 488 (12 064)	67	72% (15%)	155
High	3.5 (0.7)		55% (11%)		$11 162 (4386)		53% (16%)	
Medium	3.7 (1.2)		67% (27%)		$43 008 (10 007)		84% (17%)	
Low	2.5 (0.5)		22% (10%)		$1479 (326)		67% (16%)	

^a^A rating scale between 0 and 10 was used with 10 being the highest risk

^b^On average, respondents reported 99% (SE 7%) out-of-pocket in Anchorage and 89% (SE 7%) in Fairbanks with the remainder covered by insurance. An outlier of $250 000 was removed for calculating the mean

Fairbanks homeowners’ perceived risk of permafrost thaw was almost twice (3.4) that of Anchorage homeowners (1.9) on the ten-point subjective risk-rating scale (Table 4). Most Fairbanks homeowners (72%) believed that the permafrost hazard worsened over the past decade, compared to 31% in Anchorage.

**Fig. 6:**
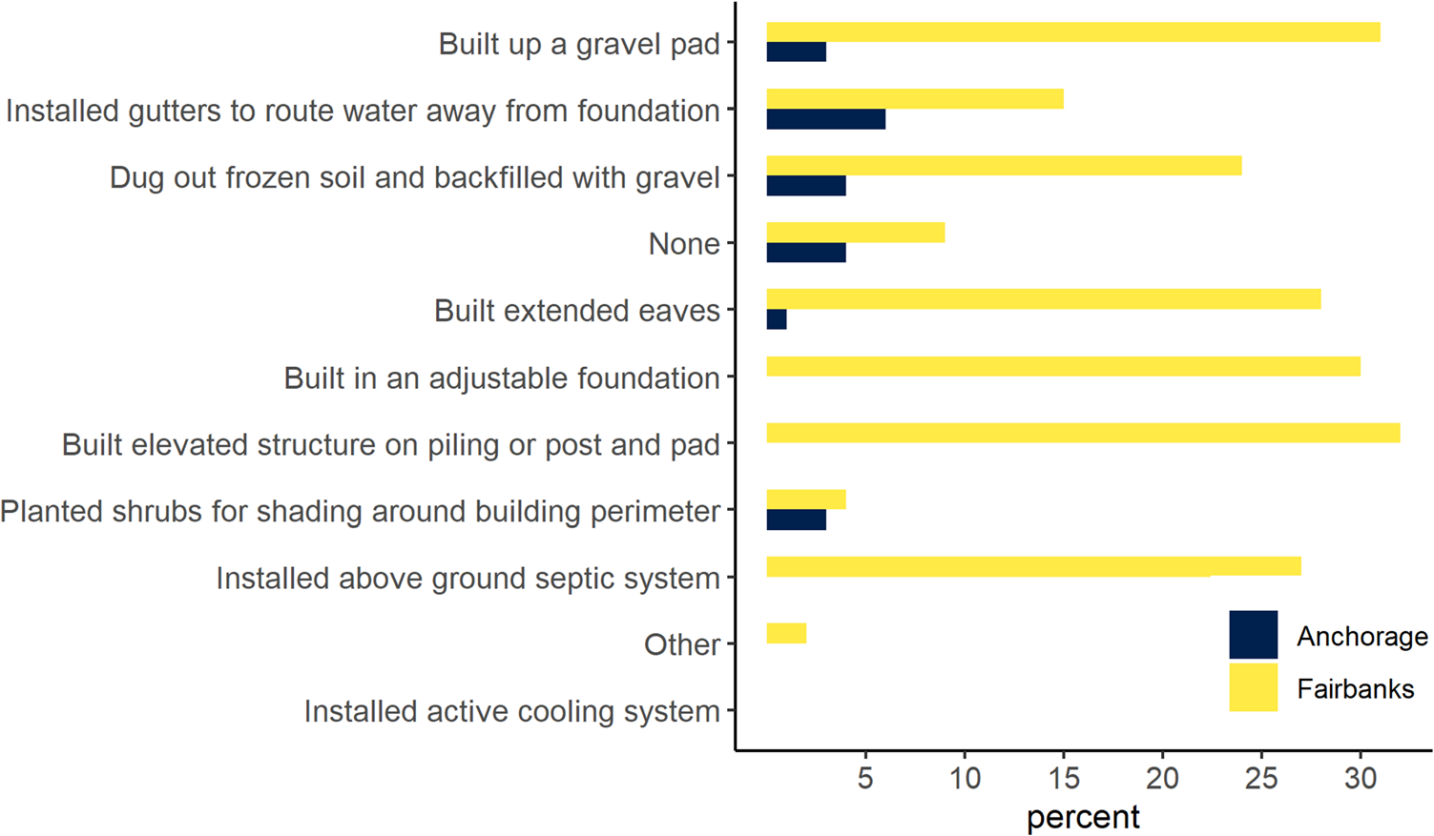
Estimated percentage of homeowners who reported to have been affected by permafrost and completed permafrost mitigation on their property, Anchorage *n* = 23, Fairbanks *n* = 67

Fairbanks homeowners reported to have mitigated permafrost hazards on their property with a range of actions, whereas Anchorage homeowners were less likely to need mitigation (Fig. 6). For example, about a third (32%) of Fairbanks homeowners built an elevated structure on pilings or post and pad and 31% built up a gravel pad, compared to 0% and 1% in Anchorage, respectively. More than a quarter (27%) of Fairbanks homeowners mentioned installing an above ground septic system^1^ compared to 3% in Anchorage. Thirty percent of homeowners in Fairbanks built an adjustable foundation^2^ and 28% extended eaves to provide shading for the foundation. In Fairbanks, 2% of homeowners undertook other mitigation actions including snow removal to keep ground frozen,^3^ installing plastic liners and improving drainage to keep soil dry around the house, and installing the foundation on 60ft pilings. None of the Anchorage respondents reported having installed an adjustable foundation, pilings, or an above ground septic system. Only one respondent reported to have installed thermosyphons to keep permafrost frozen around the basement slab. The mean reported mitigation costs associated with the entire time respondents lived in their residences equaled $3717 for Anchorage homeowners and was ten times larger in Fairbanks equaling $31 488. For the current housing stock, total private permafrost mitigation costs were estimated at $19.9 million (SE 14.8 million) for Anchorage and $226.3 million (SE 185.4 million) for Fairbanks. Less than 1% of homeowners in Anchorage and Fairbanks had homeowners’ insurance that covered damages from permafrost.

For Fairbanks, we estimated that 44% of residents observed ground subsidence or sink holes compared to 8% in Anchorage. Of those who saw ground subsidence or sink holes, 91% of Fairbanks respondents believed that these features were somewhat likely or very likely related to permafrost compared to 57% in Anchorage. In Fairbanks, 33% of respondents were aware of permafrost on their property or next to it, only 9% in Anchorage. Of those who were not aware of permafrost on their property, 4% were concerned and 16% thought permafrost possibly affected their property.

## Discussion

Urban residents in Alaska are increasingly affected by multi-hazard environments having significant financial and health-related impacts. The range of mitigation actions across cities were associated with differences in the hazards and environmental conditions and social and economic characteristics. Private mitigation action across all three hazards is significant and generally congruent to the assessed hazard levels, reported impacts, and perceived risks (Tables 2, 3, 4). For example, wildfire insurance coverage was 63% in Fairbanks and 29% in Anchorage, matching the estimated percentage of wildfire affected homeowners in Fairbanks (58%) and Anchorage (29%). The estimated 63% of homes with wildfire coverage in Fairbanks may seem low and may indicate that a significant proportion of homes are not insured. For homeowners seeking wildfire coverage outside fire service areas; i.e., residing in an area not served by a fire department supported with local tax revenue, separate wildfire policies may be required. In Fairbanks, 7% of residential properties are outside the fire service area. Even though land tends to be more affordable in this area, the additional insurance costs may become unaffordable for many lower-income households attracted to these areas.

Overall, the results suggest residents are proactive and engaged in protective action against climate-related hazards. Taking preemptive action to mitigate climate stressors has been shown to be linked to people’s direct experiences with these hazards (Pulwarty and Melis 2001; Page and Dilling 2020). Other research also showed that adaptation may be more challenging with climate-related hazards that are changing more rapidly in intensity and frequency, consistent with most respondents indicating worsening hazards (Tables 2, 3, 4) (Glaas et al. 2015).

On all three hazards, a much higher percentage of Fairbanks residents believe that each of the three hazards has worsened in the past decade, a result supported by a much higher percentage of homeowners being affected by all three hazards in Fairbanks compared to Anchorage (Table 1). Results in Table 2 were consistent with higher wildfire danger in the Interior of Alaska partly driven by a higher chance of lightning strikes igniting wildfires in this region, a drier climate, and a more clustered population in the Fairbanks wildland urban interface (Grabinski and McFarland 2020; Chen et al. 2021). Also, the frequency and area burned is increasing Alaska wide, largely driven by changes in Interior Alaska, while Anchorage has not seen a major increase in acres burned in recent decades. Similarly, survey results on permafrost were consistent with the literature on permafrost extent in Fairbanks where permafrost with significant ground ice content is more widely distributed compared to Anchorage (Table 4) (Jorgenson et al. 2006). The low percentage of Anchorage residents reporting permafrost hazard impacts (15%) is consistent with existing permafrost conditions affecting relatively few homeowners compared to Fairbanks. Consequently, the estimated mean permafrost mitigation cost in Fairbanks was more than eight times the mean mitigation cost in Anchorage and the percentage of affected homeowners was 36% higher in Fairbanks. Respondents perceived risk was also consistent with these results.

The risk associated with surface ice hazards was much higher in Anchorage, illustrated by mean damage estimates twice as high in Anchorage and the percentage of homeowners with damages 17% higher compared to Fairbanks (Table 3). This result was consistent with a higher likelihood of freezing rain and overall, a milder climate in Anchorage compared to Fairbanks (Bieniek et al. 2018; Pan et al. 2018; NOAA 2023). However, Fairbanks residents had higher perceived risk, higher mean mitigation expenses, and a higher percentage of homeowners’ believing that surface ice hazards have worsened in the past decade. This result may indicate that the number of icing events in Fairbanks are increasing and that residents are more risk averse towards this hazard, also supported by the higher mean mitigation costs compared to the mean damages (Table 3).

There are several possible explanations for this result. First, in 2021–2022, Fairbanks experienced an unusually mild winter with large precipitation, some of it falling as rain and causing widespread infrastructure impacts and service interruptions (Walsh et al. 2022).^4^ This significant icing event occurred over the larger Fairbanks area, affecting the entire population. In addition, research has shown people’s risk perceptions are affected by extreme events and emotions that differ from the actual risk (Slovic and Weber 2013). The uncommon weather events may have made residents more aware of the surface ice hazard and predictions are consistent with widespread perceived worsening of this hazard in Fairbanks (Table 3) (Pan et al. 2018). Second, we find some evidence of risk compensation (Peltzman 1975), suggesting that the higher use of studded tires in Anchorage, for example, may result in more risk-taking behavior as illustrated by higher damages and lower perceived risk and lower mitigation costs in Anchorage. Third, differences in the perceived risk rating for the surface ice hazard are consistent with the differences in mitigation expenses between the two cities, suggesting higher mitigation behavior is consistent with higher perceived risk (Masson et al. 2020).

Perceived risk estimates also showed some inconsistencies with the surface ice and permafrost hazards (Tables 3, 4). For example, in the permafrost case, perceived risk by residents living in the medium hazard zone compared to the high hazard zone, was 0.8 points higher in Anchorage and 0.2 points higher in Fairbanks. Similarly, subjective risk ratings for surface ice hazards in the medium hazard zone compared to the high hazard zone were 0.4 points higher in Anchorage and 0.6 points higher in Fairbanks. We consider these deviations to be within the variance associated with the means and partly attribute the discrepancies to heuristics and biases that arise during attempts to assign numerical values to rather abstract concepts (Tversky and Kahneman 1974). Also, perceived risks were reported to be significantly lower for residents living in the low hazard zones of these two hazards, supporting the overall consistency of the hazard assessments with actual risk.

The high (95%) support for preemptively shutting off electricity during high wildfire danger was consistent with assessed wildfire hazards in Fairbanks. In contrast, the most support (95%) for this policy in Anchorage was in the low wildfire hazard zone where utility shut offs are less likely to occur, and less likely to inconvenience residents (Table 2). Due to the politically controversial issue of this policy (Mildenberger et al. 2022), the above result in Anchorage is likely due to political leanings and might have less to do with actual risk.

The raking approach likely reduced but did not eliminate bias in the mitigation and damage estimates. The raking algorithm tried to find weights that are a compromise between aligning the sample’s income variable with the marginal distribution found in the population and at the same time calibrating the sampling proportions to reflect the population proportions across strata (Pike 2007). In our case, household income served only as a proxy for the unknown distribution of income among homeowners, a wealthier subset of each city’s residential population. In addition, age could have been used for raking, but since it is measured at the individual level, household income was preferred. Age was also positively correlated with income.

In Anchorage, the survey had disproportionally higher response rates in the high wildfire and high surface ice hazard zones with fewer responses in the high permafrost hazard zone (Supplementary Information 1 Table S4). In Fairbanks, response in the high wildfire and in the high permafrost zones was disproportionally higher, as these zones are more likely being occupied by lower-income homeowners who might be less inclined to conduct costly mitigation, especially for the permafrost hazard (Fig. 2). Thus, permafrost mitigation costs are considered a lower bound estimate, not accounting for changes in living conditions associated with permafrost damage to private homes and not accounting for non-respondents who abandoned their properties due to permafrost thaw (Ward Jones et al. 2022). In addition, the results are an underestimate and are solely associated with homeowners amounting to 63% of the Anchorage and 59% of the Fairbanks population (U.S. Census 2021). Renters who are also more likely lower-income households are not included in these estimates but may have less incentive to mitigate hazards on the properties they reside in.

Increased climate-related impacts on lower-income households residing in high hazard zones are consistent with recent research that found that the impacts of climate change are disproportionally distributed across the American public with higher impacts on vulnerable populations such as lower-income households, with exceptions (EPA 2021). Specifically, we found that the observed patterns of hazard impacts across income quartiles were in part also associated with varying property values. For example, in Fairbanks, land selection by lower-income homeowners is partly driven by the higher affordability and availability of land in the high permafrost and high wildfire hazard zones, largely outside fire service areas. In Anchorage, however, the opposite is true. The high wildfire zone on the city’s eastern edge (Supplementary Information 1 Fig. S13) is associated with views and consequently higher property values attracting higher-income households (Fig. 2).

### Policy recommendations

As the impacts of wildfire, surface ice, and permafrost thaw are predicted to increase in northern latitudes, and human populations want to expand into hazard prone areas (Walsh et al. 2020; Ward Jones et al. 2022), climate risk mitigation and adaptation are essential components for risk management and urban planning (Dhar and Khirfan 2017). Government-funded mitigation programs that work closely with private homeowners, such as the *Firewise* program, are a first line of defense against increasing hazards, raising awareness, and incentivizing private action (McCaffrey 2015). However, the challenges of multi-hazard environments also warrant these programs to broaden their focus and become more sophisticated.

Mitigating hazards in zones where multiple hazards occur and where mitigation actions may result in unintended consequences, deserve careful mitigation planning. For example, in the high wildfire and high permafrost hazard zone, affecting approximately 1400 homeowners in the study area, the permafrost mitigation action of planting shrubs for shading around the building perimeter may work against the creation of defensible space aimed at mitigating wildfire hazards. In such more complex mitigation situations, education and outreach are important to inform homeowners about the trade-offs between mitigation actions. Specific mitigation programs with knowledgeable personnel such as through the *Firewise* program in Alaska can also provide more customized solutions that are location specific. For example, both permafrost thaw and wildfire hazards can be mitigated by replacing conifers with fire-resistant vegetation such as larch, providing shade, and insulating below grade around building perimeters instead of planting shrubs.

Local participation in preventive mitigation of climate-related hazards supports and enhances the broader community-based response and benefits not only private homeowners but more broadly disaster and risk management in general (Cutter et al. 2012). Private mitigation action also serves as an important tool to reduce risk for homeowners and insurance companies alike, ensuring long-term viability of the industry by minimizing moral hazard (Arrow 1971). However, we found that most wildfire insurance does not require or incentivize homeowners to prepare properties for wildfire while refusing to cover properties in zones with high wildfire hazard. This result may imply that insurance companies and homeowners may benefit from more accurate information related to hazard assessments and human mitigation behavior as provided by this study.

Insurance coverage alone, however, cannot be considered sufficient risk management. Broad population-wide mitigation action requires proper incentives to encourage preventative action. Public mitigation programs such as *Firewise* can incentivize mitigation action and increase public engagement and awareness. Research suggests that outreach and education programs can also build community capacity, increase information sharing, and foster mitigation activities (McGee 2011; Stidham et al. 2014). However, culture and local context are important considerations to implement effective programs (Christianson et al. 2014). Participation should be tailored to those in high hazard areas, especially lower-income homeowners and those who may not be reached in these high hazard zones. Public programs incentivizing mitigation can also collect valuable information for integrating local knowledge in disaster preparedness, optimizing response at all levels of governance, and empowering local decision making (Griego et al. 2020). Most importantly, these programs empower people to respond to environmental risk, make their actions affordable, and result in higher mitigation success consequently reducing risk and cost for homeowners and insurance companies alike.

Besides improvements in outreach and education covering multi-hazard environments and incentivizing mitigation among lower-income households, local governments also play an important role. Local institutional structures and policies vary across communities, often requiring innovative approaches to implementing mitigation programs (McCaffrey 2015; Madsen et al. 2018). For example, the Municipality of Anchorage is a “home rule” borough (county) form of government, with the maximum level of self-government, and authority to tax and establish programs. In contrast, the Fairbanks North Star Borough is a Second Class Borough with limited powers, where residents grant permission to establish programs. The Borough contains two incorporated cities, Fairbanks and North Pole, with more regulatory powers.

Anchorage has established an active sub-local government of 37 Community Councils for assisting the Anchorage Assembly with communication, public process, and neighborhood issues. Several of these Community Councils in the high wildfire zone are active in *Firewise*. The Community Councils provide awareness and specificity necessary for fire prevention and planning at a micro landscape and neighborhood level. The sub-local government level in Fairbanks consists of 103 Road Service Areas and five Fire Service Districts. Each service area was established at the request of residents and authorized by the Fairbanks North Star Borough. Unfortunately, many roads in these service areas do not conform to standard width and condition considered wildfire egress. Nevertheless, the service areas could be an effective level of governance for wildfire prevention in Fairbanks and other areas with similar governance structure. Because of their direct connection to the people they serve, sub-level forms of local government, such as community councils or service areas provide effective ways to advise local governments in their planning and implementation of hazard mitigation actions and development of zoning regulations that discourage building in high hazard zones.

## Conclusion

This study contributes to a better understanding of urban multi-hazard environments, especially in northern latitudes, emphasizing the importance of assessing location-specific impacts on human populations and incentivizing preemptive hazard mitigation. The study investigated patterns of human hazard response and impacts of wildfire, surface ice, and permafrost. The interplay of multi-hazard environments within social-ecological systems remains an important area for future research. As climate-related hazards are increasing in intensity and frequency, effective human response at all levels requires specific information about impacts and mitigation response across hazards and population characteristics that highly vary by geography. This study found that lower-income households are disproportionally affected by climate-related hazards, especially high permafrost and high wildfire hazards. Improving effective public outreach, incentivizing homeowners to take mitigation action, and implementing effective and comprehensive programming through existing local government structures are key to long-term community-wide risk reduction and climate justice.

### Supplementary Information

Below is the link to the electronic supplementary material.

Supplementary file1 (PDF 2,601 KB)

Supplementary file2 (PDF 93 KB)

## Footnotes

1 Fairbanks has a high proportion of dry cabins with outhouses and some respondents could have indicated to have “above ground septic systems” when in fact they had outhouses.

2 Homes built on gravel pads have likely also adjustable foundations as these two actions are often combined, resulting in similar proportions for the two mitigation actions.

3 Snow removal around foundations keeps the foundation cooler, taking away snow which acts as an insulating layer for summer heat remaining in the ground longer.

4 There have been multiple impactful rain-in-winter events in recent years, including the severe Thanksgiving week rain in November 2010 and rain events severe enough to cause school closures in January and November 2013 and February 2015.
