# Supplementary material for: Increasing multi-hazard climate risk and financial and health impacts on northern homeowners
Source: Ambio. 2023 Nov 13;53(3):389–405. doi: 10.1007/s13280-023-01951-z (PMC10837396; doi:10.1007/s13280-023-01951-z)
Supplement: Supplementary file 1 — Supplementary file1 (PDF 2,601 KB) [file 13280_2023_1951_MOESM1_ESM.pdf]

**Ambio**

Supplementary Information 1

# Increasing multi-hazard climate risk and financial and health impacts on northern homeowners

Tobias Schwoerer [a*], Jennifer I. Schmidt [b], Matthew Berman [b], Peter Bieniek [a], Louise M. Farquharson [c] Dmitry Nicolsky [c], James Powell [d], Rachel Roberts [b], Rick Thoman [a], Robert Ziel [c]

[a] International Arctic Research Center, University of Alaska Fairbanks, PO Box 75734, Fairbanks, AK 99775-7340, USA,
[b] Institute of Social and Economic Research, University of Alaska Anchorage, 3211 Providence Dr. Anchorage, AK 99509, USA
[c] Geophysical Institute, University of Alaska Fairbanks, PO Box 757320, Fairbanks, AK, 99775-7340, USA
[d] Alaska Coastal Rainforest Center, University of Alaska Southeast, 11066 Auke Lake Way, Juneau, AK 99801, USA

* corresponding author: tschwoerer@alaska.edu, +1 (907) 786 5404

# 1) Information on sampling and sampling frame

**Table S1** Sampling proportions for Anchorage sample

n= 2000

| | | Stratification | | | | | | | | |
|---|---|---|---|---|---|---|---|---|---|---|
| | | Fire | | | | | | | | |
| | | High | | | Medium | | | Low | | |
| Permafrost | | High | Medium | Low | High | Medium | Low | High | Medium | Low |
| Ice | High | 1 | 4 | 7 | 10 | 13 | 16 | 19 | 22 | 25 |
| | Medium | 2 | 5 | 8 | 11 | 14 | 17 | 20 | 23 | 26 |
| | Low | 3 | 6 | 9 | 12 | 15 | 18 | 21 | 24 | 27 |

Number of Parcels in Each Stratification

| | | fire | | | | | | | | | |
|---|---|---|---|---|---|---|---|---|---|---|---|
| | | High | | | Medium | | | Low | | | Total |
| Permafrost | | High | Medium | Low | High | Medium | Low | High | Medium | Low | |
| ice | High | 0 | 5 | 3039 | 1113 | 6290 | 10224 | 0 | 0 | 1154 | 21825 |
| | Medium | 20 | 16 | 1980 | 1755 | 13494 | 13534 | 162 | 0 | 3716 | 34677 |
| | Low | 16 | 4 | 2224 | 336 | 2813 | 14826 | 0 | 0 | 5427 | 25646 |
| | Total | 36 | 25 | 7243 | 3204 | 22597 | 38584 | 162 | 0 | 10297 | 82148 |

Percentage of Parcels in Each Stratification

| | | fire | | | | | | | | | |
|---|---|---|---|---|---|---|---|---|---|---|---|
| | | High | | | Medium | | | Low | | | Total |
| Permafrost | | High | Medium | Low | High | Medium | Low | High | Medium | Low | |
| ice | High | 0.00% | 0.01% | 3.70% | 1.35% | 7.66% | 12.45% | 0.00% | 0.00% | 1.40% | 26.57% |
| | Medium | 0.02% | 0.02% | 2.41% | 2.14% | 16.43% | 16.48% | 0.20% | 0.00% | 4.52% | 42.21% |
| | Low | 0.02% | 0.00% | 2.71% | 0.41% | 3.42% | 18.05% | 0.00% | 0.00% | 6.61% | 31.22% |
| | Total | 0.04% | 0.03% | 8.82% | 3.90% | 27.51% | 46.97% | 0.20% | 0.00% | 12.53% | 100.0% |

| | | Proportional Number of Parcels | | | | | | | | | |
|---|---|---|---|---|---|---|---|---|---|---|---|
| | | Fire | | | | | | | | | |
| | | High | | | Medium | | | Low | | | |
| Permafrost | | High | Medium | Low | High | Medium | Low | High | Medium | Low | |
| Ice Risk | High | 0 | 0 | 74 | 27 | 153 | 249 | 0 | 0 | 28 | |
| | Medium | 0 | 0 | 48 | 43 | 329 | 330 | 4 | 0 | 90 | |
| | Low | 0 | 0 | 54 | 8 | 68 | 361 | 0 | 0 | 132 | |
| | | | | | | | | | | | 2000 |

**Table S2** Sampling proportions for Fairbanks sample

n= 2000

| | | Fire hazard | | | | | | | | | |
|---|---|---|---|---|---|---|---|---|---|---|---|
| | | High | | | Medium | | | Low | | | Total |
| Permafrost | | High | Medium | Low | High | Medium | Low | High | Medium | Low | |
| Ice Risk | High | 676 | 275 | 1596 | 746 | 8110 | 590 | 0 | 0 | 0 | 11993 |
| | Medium | 729 | 138 | 1058 | 97 | 1920 | 1023 | 7 | 0 | 6 | 4978 |
| | Low | 93 | 43 | 308 | 184 | 157 | 318 | 0 | 0 | 0 | 1103 |
| | Total | 1498 | 456 | 2962 | 1027 | 10187 | 1931 | 7 | 0 | 6 | 18074 |

| | | Fire hazard | | | | | | | | | |
|---|---|---|---|---|---|---|---|---|---|---|---|
| | | High | | | Medium | | | Low | | | Total |
| Permafrost | | High | Medium | Low | High | Medium | Low | High | Medium | Low | |
| Ice Risk | High | 3.74% | 1.52% | 8.83% | 4.13% | 44.87% | 3.26% | 0.00% | 0.00% | 0.00% | 66.35% |
| | Medium | 4.03% | 0.76% | 5.85% | 0.54% | 10.62% | 5.66% | 0.04% | 0.00% | 0.03% | 27.54% |
| | Low | 0.51% | 0.24% | 1.70% | 1.02% | 0.87% | 1.76% | 0.00% | 0.00% | 0.00% | 6.10% |
| | Total | 8.29% | 2.52% | 16.39% | 5.68% | 56.36% | 10.68% | 0.04% | 0.00% | 0.03% | 100.0% |

| | | Stratification | | | | | | | | |
|---|---|---|---|---|---|---|---|---|---|---|
| | | Fire hazard | | | | | | | | |
| | | High | | | Medium | | | Low | | |
| Permafrost | | High | Medium | Low | High | Medium | Low | High | Medium | Low |
| Ice Risk | High | 1 | 4 | 7 | 10 | 13 | 16 | 19 | 22 | 25 |
| | Medium | 2 | 5 | 8 | 11 | 14 | 17 | 20 | 23 | 26 |
| | Low | 3 | 6 | 9 | 12 | 15 | 18 | 21 | 24 | 27 |

| | | Fairbanks, n | | | | | | | | |
|---|---|---|---|---|---|---|---|---|---|---|
| | | Fire | | | | | | | | |
| | | High | | | Medium | | | Low | | |
| Permafrost | | High | Medium | Low | High | Medium | Low | High | Medium | Low |
| Ice Risk | High | 75 | 30 | 177 | 83 | 897 | 65 | 0 | 0 | 0 |
| | Medium | 81 | 15 | 117 | 11 | 212 | 113 | 1 | 0 | 1 |
| | Low | 10 | 5 | 34 | 20 | 17 | 35 | 0 | 0 | 0 |

## 2) Additional tables

**Table S3** Respondent sample characteristics by city

| Variable | Anchorage | Fairbanks |
|---|---|---|
| Respondent count | 320 | 379 |
| Mean annual homeowner income in U.S. $ (SD) | $127 823 (64 233) | $116 257 (56 675) |
| Mean respondent age in years (SD) | 54.8 (14.5) | 56.4 (14.2) |
| Mean homeowner size in persons (SD) | 2.6 (1.4) | 2.6 (1.3) |
| Mean city residency in years (SD) | 15.7 (10.8) | 18.2 (11.2) |
| Homeowners in % | 99.4% | 97.1% |
| Homeowners with children under 18 in % | 28% | 27% |

**Table S4** Comparing the sample with population proportions in each of the hazard zones

| | | | Anchorage | | | Fairbanks | | |
|---|---|---|---|---|---|---|---|---|
| Wildfire | Ice | Permafrost | Population proportion | Response proportion | Response count | Population proportion | Response proportion | Response count |
| High | High | High | | | | 3.7% | 2.9% | 11 |
| High | High | Low | 3.7% | 4.4% | 14 | 9.0% | 10.6% | 40 |
| High | High | Medium | | | | 1.5% | 1.3% | 5 |
| High | Low | High | | | | 0.5% | 0.8% | 3 |
| High | Low | Low | 2.7% | 3.4% | 11 | 1.7% | 1.1% | 4 |
| High | Low | Medium | | | | 0.3% | 0.5% | 2 |
| High | Medium | High | | | | 4.0% | 5.5% | 21 |
| High | Medium | Low | 2.4% | 3.1% | 10 | 6.2% | 8.4% | 32 |
| High | Medium | Medium | | | | 0.8% | 1.3% | 5 |
| Medium | High | High | 1.4% | 0.6% | 2 | 4.1% | 4.7% | 18 |
| Medium | High | Low | 12.5% | 16.3% | 52 | 3.2% | 5.0% | 19 |
| Medium | High | Medium | 7.7% | 7.5% | 24 | 44.7% | 39.8% | 151 |
| Medium | Medium | High | 2.2% | 2.2% | 7 | 1.0% | 0.8% | 3 |
| Medium | Medium | Low | 16.5% | 17.5% | 56 | 1.7% | 1.6% | 6 |
| Medium | Medium | Medium | 16.5% | 15.9% | 51 | 0.9% | 0.5% | 2 |
| Medium | Low | High | 0.4% | 0.6% | 2 | 0.6% | 0.5% | 2 |
| Medium | Low | Low | 18.1% | 19.4% | 62 | 5.6% | 6.3% | 24 |
| Medium | Low | Medium | 3.4% | 4.1% | 13 | 10.5% | 8.2% | 31 |
| Low | High | Low | 1.4% | 0.3% | 1 | | | |
| Low | Low | Low | 6.8% | 2.2% | 7 | | | |
| Low | Medium | Low | 4.5% | 2.5% | 8 | [a] <1% | 0% | n/a |
| Low | Medium | High | [a] <1% | 0% | n/a | [a] <1% | 0% | n/a |

a) A total of 4 parcels in Anchorage and a total of 5 parcels in Fairbanks fall into these hazard zones and did not receive any responses.

**Table S5** Estimated percentage of homeowners affected by wildfire

| Type of impact | Anchorage n=61 | Fairbanks n=141 |
|---|---|---|
| Had to reduce outdoor activity | 13% | 48% |
| Had to stay indoors due to smoke | 14% | 43% |
| Other [a)] | 5% | 16% |
| Service interruptions | 1% | 5% |
| Loss of income | <1% | 1% |
| Not affected | 80% | 42% |

a) Under other, respondents mentioned health, travel, work, and property access restrictions as well as temporary and extended evacuations.

**Table S6** Stated support for electric utilities shutting off power proactively during high fire danger

| Level of support | Anchorage % n=300 | Fairbanks % n=364 |
|---|---|---|
| Definitely yes | 22% | 31% |
| Probably yes | 37% | 48% |
| Might or might not | 22% | 13% |
| Probably not | 8% | 4% |
| Definitely not | 11% | 4% |

**Table S7** Perceptions of the government's wildfire response and preparedness

| | Effectiveness | | Preparedness | |
|---|---|---|---|---|
| Rating | Anchorage % | Fairbanks % | Anchorage % | Fairbanks % |
| Very | 52% | 76% | 40% | 72% |
| Somewhat | 21% | 17% | 35% | 19% |
| Neutral | 27% | 6% | 22% | 8% |
| Somewhat not | 1% | <1% | 2% | <1% |
| Not | <1% | 1% | 2% | <1% |
| No response | 1% | 1% | 2% | 1% |

**Table S8** Stated responsibilities for private landowners to prepare for wildfire

| Responsibility | Anchorage % | Fairbanks % |
|---|---|---|
| Participate in wildfire protection programs | 15% | 18% |
| Prepare home and landscape | 50% | 56% |
| Participate in wildfire mitigation, neighborhood, or community council efforts | 21% | 18% |
| Prepare a written wildfire plan | 5% | 4% |
| Other | 6% | 2% |
| No response | 2% | 1% |

**Table S9** Proportion of homeowners taking specific actions to mitigate ice hazards

| Mitigation action | Anchorage % | Fairbanks % |
|---|---|---|
| Spread sand and gravel | 64% | 15% |
| Purchased winter tires, studded tires, or tire chains | 64% | 19% |
| Removed hard packed snow | 62% | 10% |
| Purchased slip-resistant shoes or ice cleats | 79% | 9% |
| Spread ice melt | 64% | 5% |
| Purchased studded snow tires | 50% | 4% |
| Other | 7% | 2% |
| None | 2% | <1% |
| No response | 2% | <1% |

**Table S10** Types of vehicle tires residents use in the winter

| Type of tire | Anchorage % | Fairbanks % |
|---|---|---|
| All-season tires | 33% | 28% |
| Summer tires | 1% | 0% |
| Winter tires without studs | 23% | 52% |
| Winter tires with studs | 43% | 19% |
| No response | 0% | 0% |

**Table S11** How households learn about wildfire mitigation activities

| Source of information | Anchorage % | Fairbanks % |
|---|---|---|
| Alaska Firewise program | 14% | 40% |
| Friends | 20% | 51% |
| Neighbors | 14% | 44% |
| Community council meetings | 3% | <1% |
| Facebook and other social media | 6% | 36% |
| Community meetings and events | 7% | 5% |
| Websites | 18% | 26% |
| Radio | 17% | 26% |
| Television | 31% | 41% |
| Other [a)] | 25% | 22% |
| No response | 17% | 7% |

a) A frequent mention was newspaper, speaking to firefighters, and insurance agents.

**Table S12** Estimated number of homeowners affected by the three hazards by income and hazard levels

| Income Quartile | Hazard level | City | Wildfire | Ice | Permafrost |
|---|---|---|---|---|---|
| 1 | High | Anchorage | 5404 | 11171 | 9624 |
| 2 | High | Anchorage | 1809 | 1865 | 3081 |
| 3 | High | Anchorage | 4649 | 4311 | 5218 |
| 4 | High | Anchorage | 3182 | 2648 | 938 |
| 1 | Medium | Anchorage | 24209 | 13957 | 5746 |
| 2 | Medium | Anchorage | 5849 | 1757 | 1545 |
| 3 | Medium | Anchorage | 9433 | 3286 | 2383 |
| 4 | Medium | Anchorage | 4725 | 3039 | 1140 |
| 1 | Low | Anchorage | 560 | 5045 | 14803 |
| 2 | Low | Anchorage | 212 | 4248 | 3244 |
| 3 | Low | Anchorage | 519 | 7004 | 7000 |
| 4 | Low | Anchorage | 297 | 2517 | 6126 |
| 1 | High | Fairbanks | 1970 | 1469 | 1510 |
| 2 | High | Fairbanks | 775 | 915 | 1004 |
| 3 | High | Fairbanks | 373 | 668 | 202 |
| 4 | High | Fairbanks | 462 | 1263 | 889 |
| 1 | Medium | Fairbanks | 2156 | 1553 | 770 |
| 2 | Medium | Fairbanks | 2000 | 892 | 997 |
| 3 | Medium | Fairbanks | 893 | 331 | 210 |
| 4 | Medium | Fairbanks | 2271 | 1221 | 1203 |
| 1 | Low | Fairbanks | 31 | 1190 | 1932 |
| 2 | Low | Fairbanks | 11 | 982 | 788 |
| 3 | Low | Fairbanks | 0 | 275 | 861 |
| 4 | Low | Fairbanks | 20 | 546 | 938 |

## 3) Hazard assessment maps by hazard and city

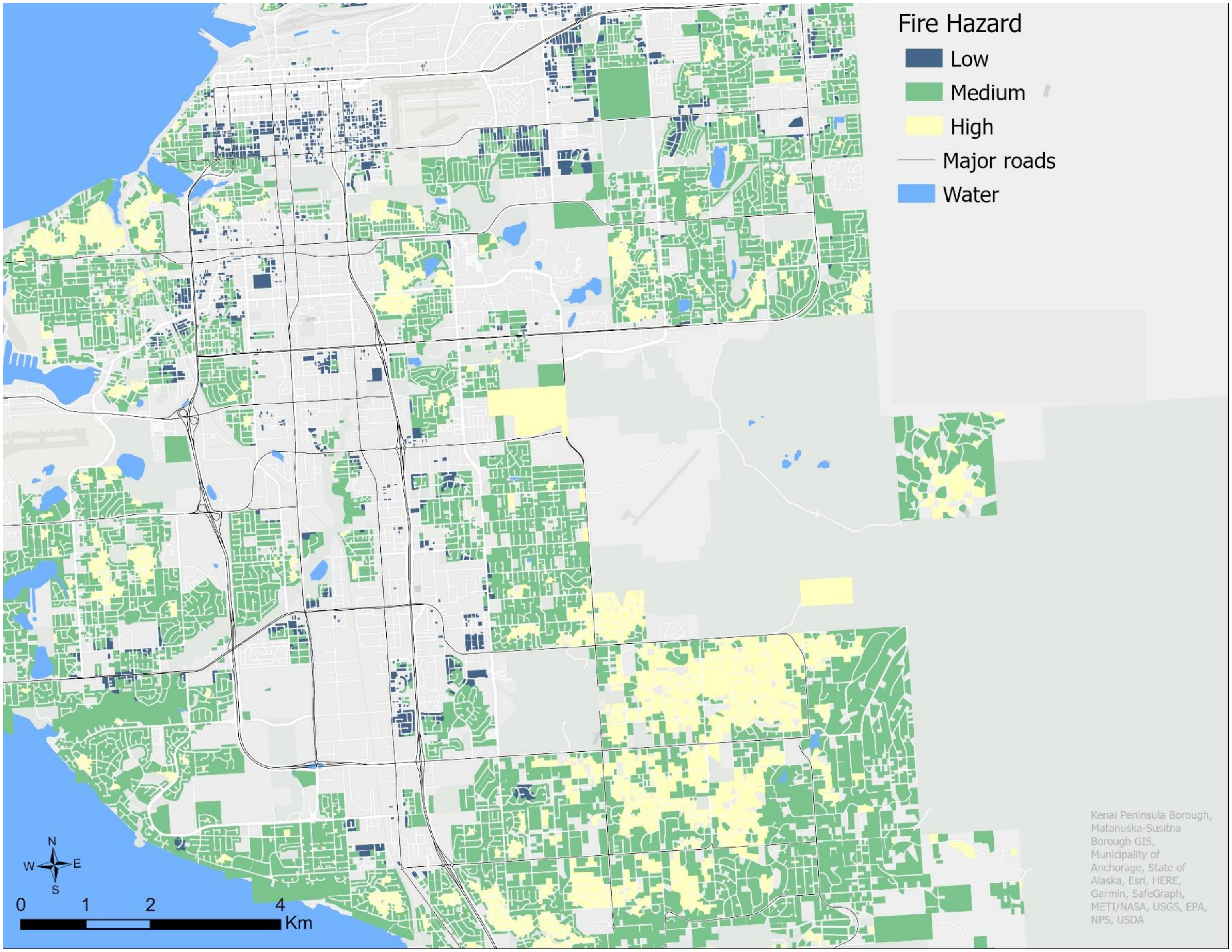


**Fig. S13** Map of assessed wildfire hazard for Anchorage

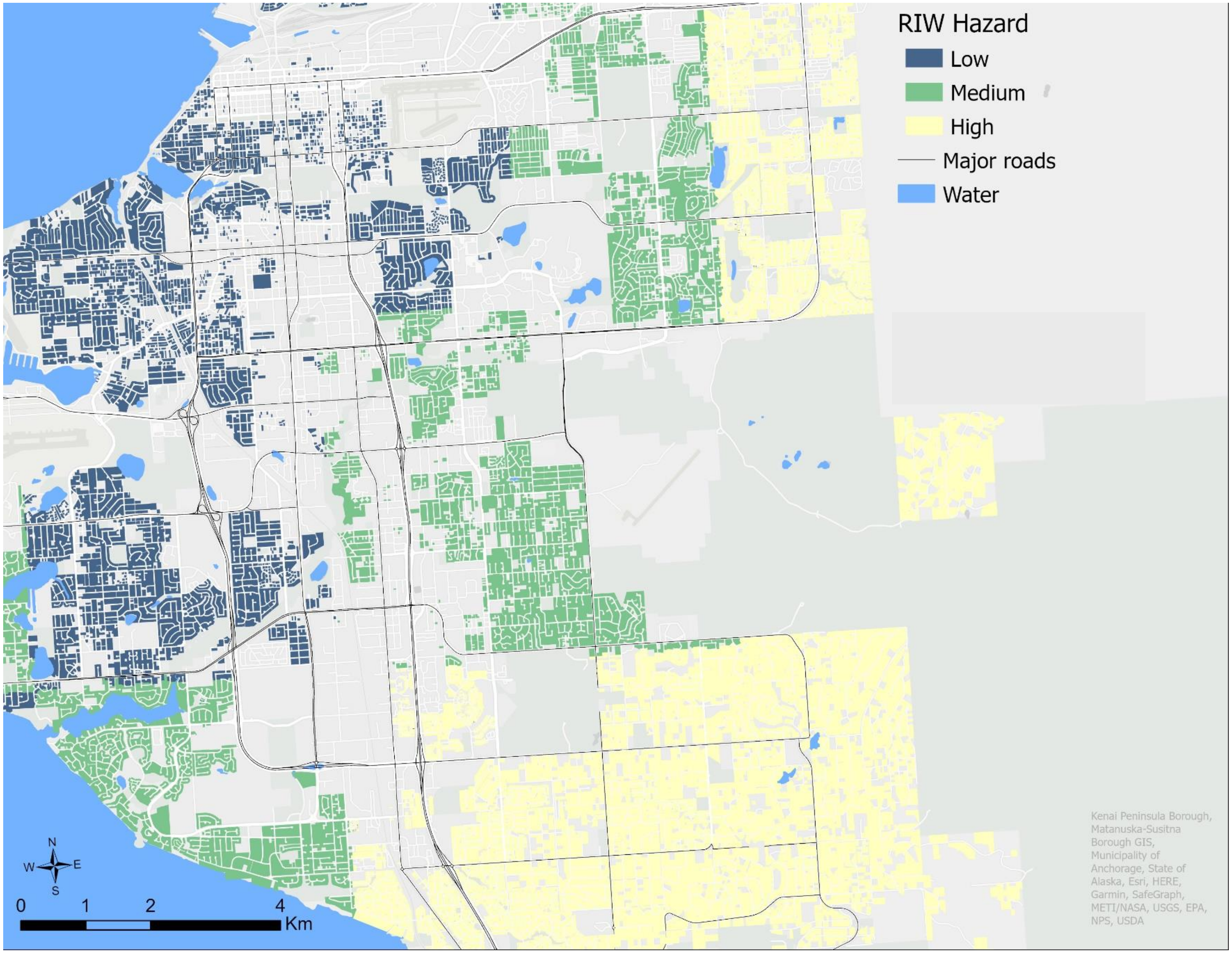


**Fig. S14** Map of assessed rain-in-winter hazard for Anchorage

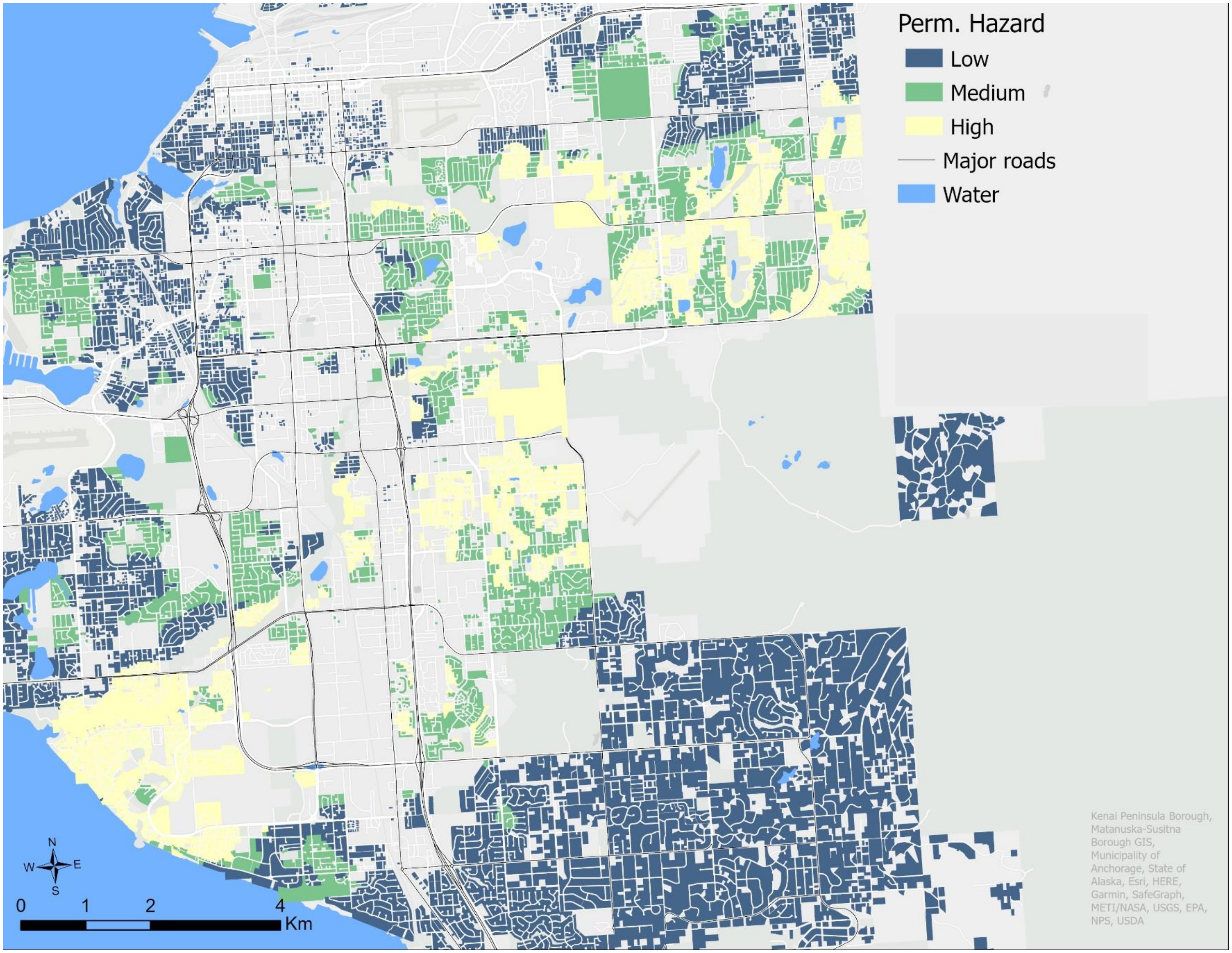


**Fig. S15** Map of assessed permafrost hazard for Anchorage

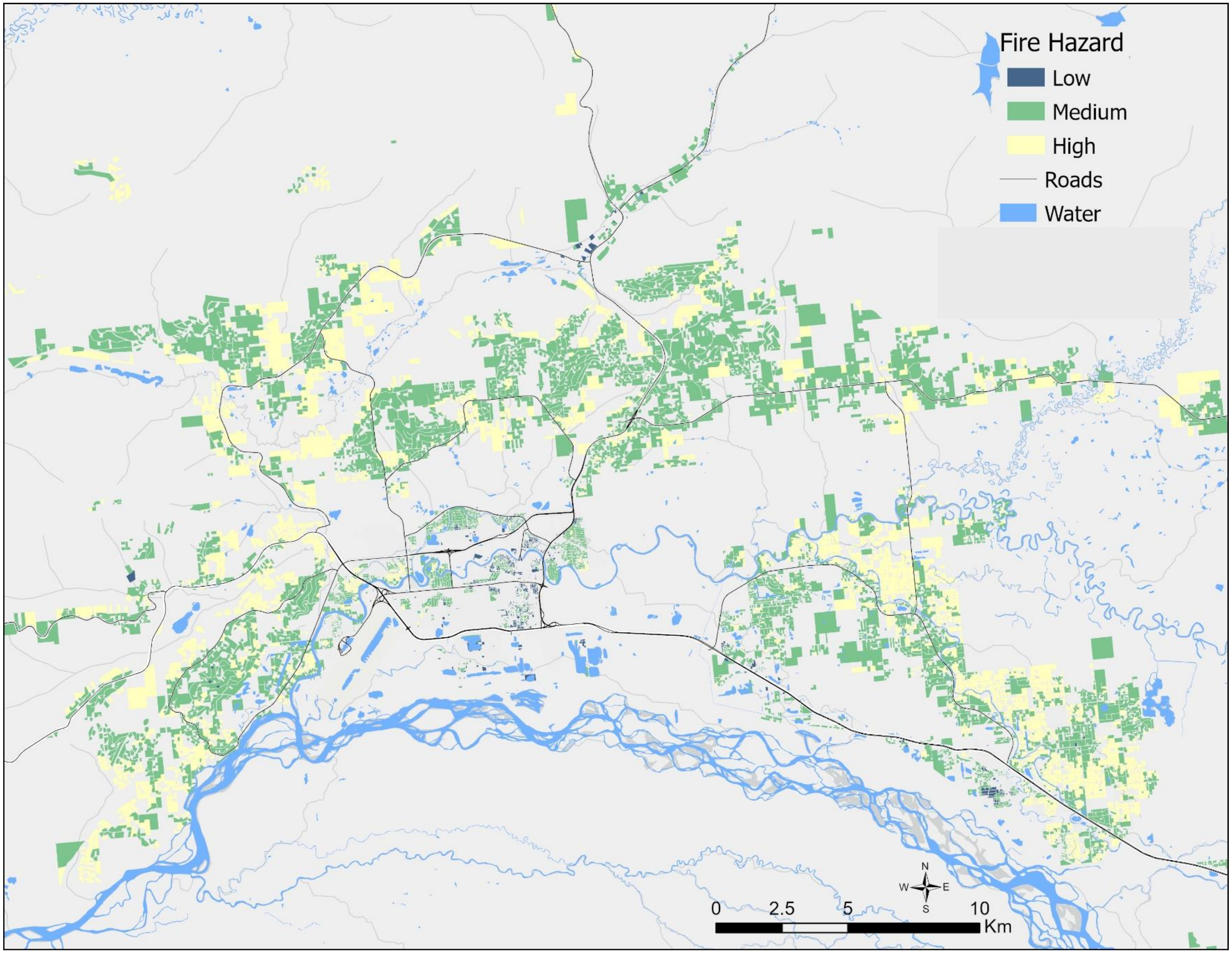

**Fig. S16** Map of assessed wildfire hazard for Fairbanks

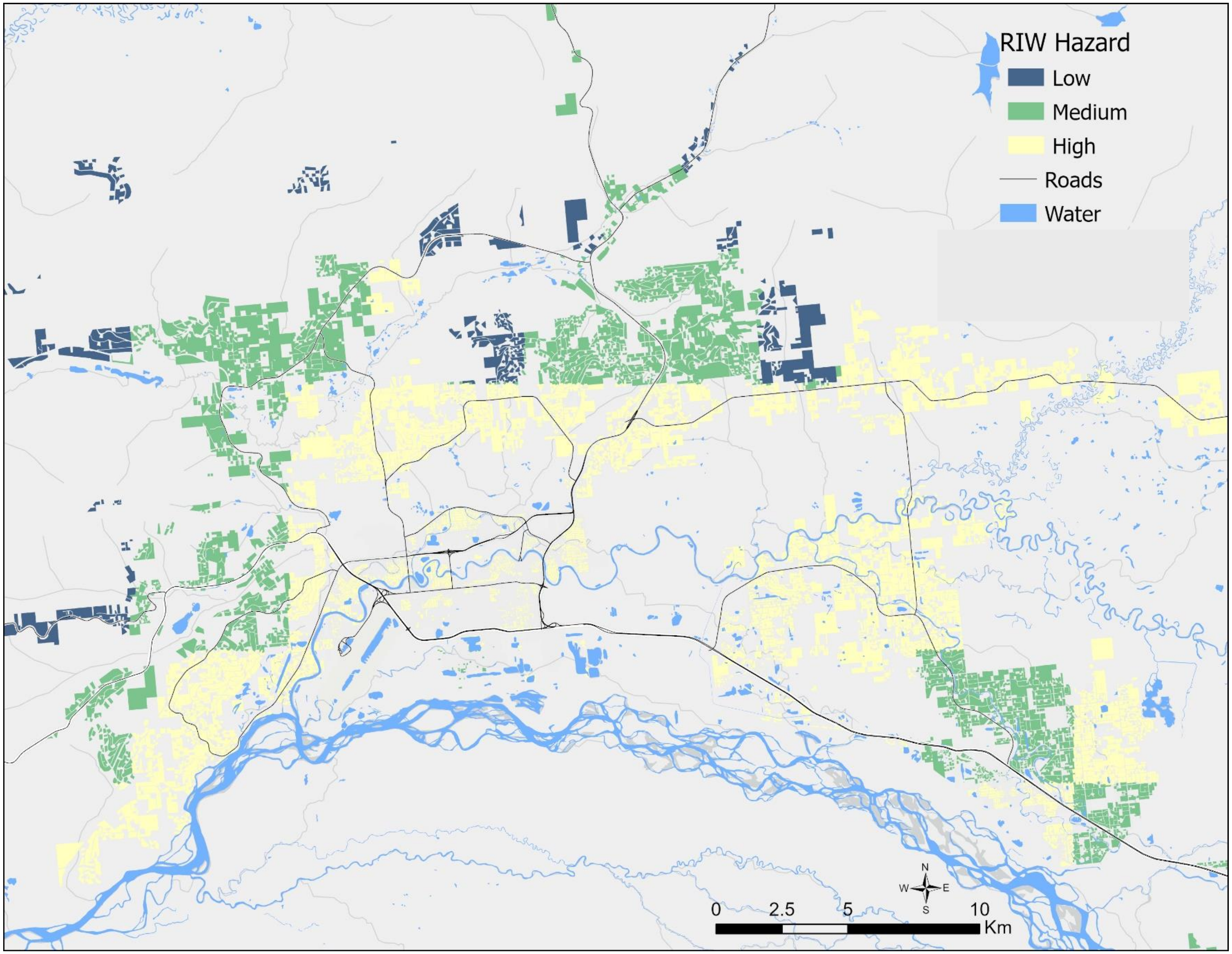


**Fig. S17** Map of assessed rain-in-winter hazard for Fairbanks

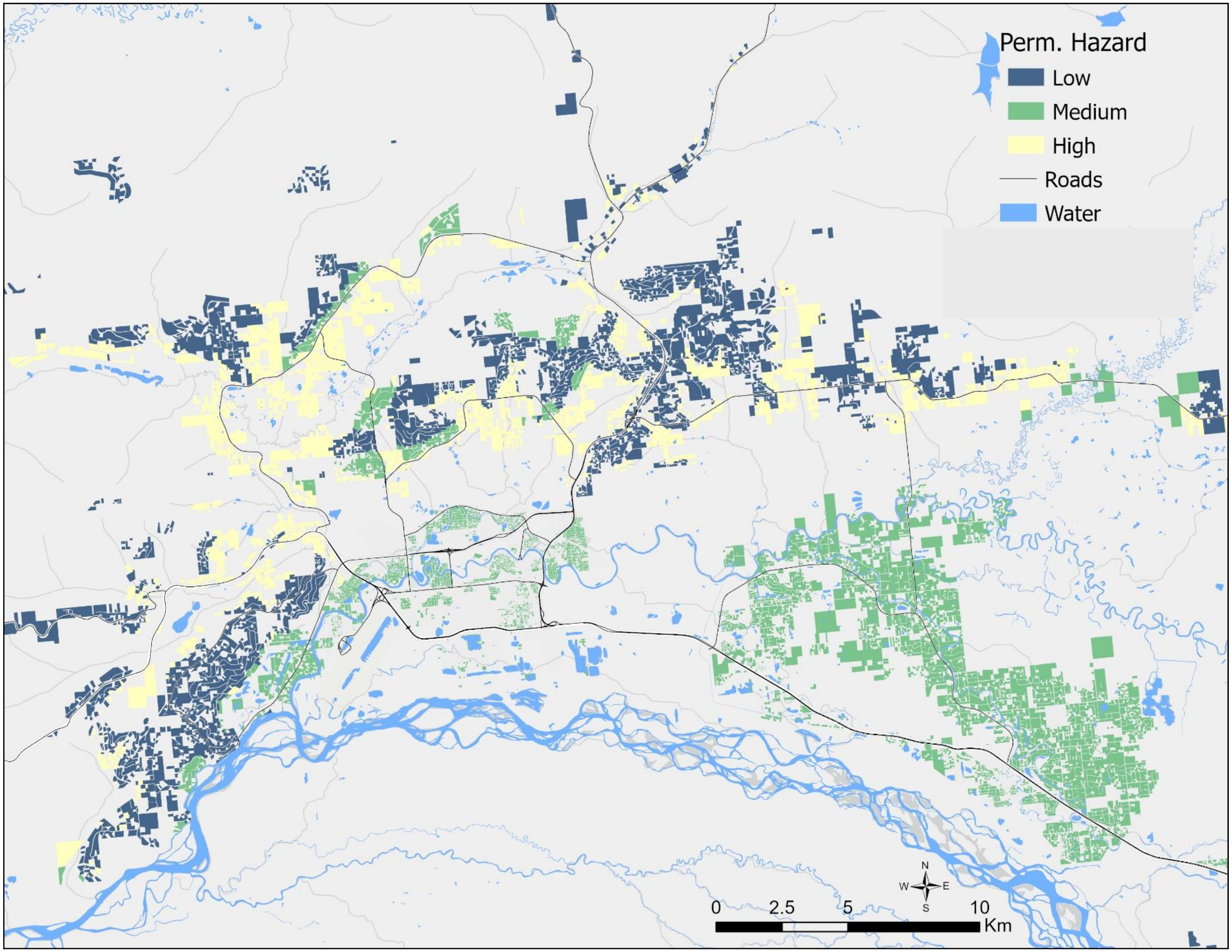


**Fig. S18** Map of assessed permafrost hazard for Fairbanks

## 4) Survey instrument

**Consent**

*Q1.*

**Multi-hazard homeowner survey**

Dear ${m://FirstName} ${m://LastName},

Thank you for participating in our study.  Your name was selected as part of a random sample obtained from property appraisals. We are University of Alaska researchers interested in better understanding people's experiences with wildfire, permafrost thaw, and ice hazards. The information you provide will inform local response to these hazards. We will share our findings with the public through reports, public presentations, and articles. Find out more about our project at respondtorisk.com.

All information you provide is voluntary and confidential, will not be associated with you, and will be matched with information from property appraisals. We will only present aggregated results and will not reveal individual information. You can stop at any time. You do not have to answer any questions that you do not want to. The survey takes 15 minutes to complete. In our first mailing, we included a $2 bill as a small thank you.
This study was reviewed by the UAF Institutional Review Board (IRB) to protect the rights and welfare of the people involved in the study. If you have questions please contact Dr. Tobias Schwoerer, Principal Investigator, Email: tschwoerer@alaska.edu or the UAF Office of Research Integrity, Email: uaf-irb@alaska.edu, Phone: 474-7800 (Fairbanks area), 1-866-876-7800 (toll-free outside Fairbanks).

Thank you for your time!

By clicking the button below, you acknowledge

**My participation in the study is voluntary.**

**I am at least 18 years of age**

**I am the head of household.**

**I am aware that I may terminate participation at any time for any reason.**

- ○ I consent, begin the survey.
- ○ I do not consent. I do not wish to participate.

*Q2.* Since you have lived at your current residence, have you been affected by any of the following environmental hazards? (Select all that apply)

- ☐ wildfire
- ☐ permafrost thaw
- ☐ ice (roads, walk ways, etc.)

*Q3.* Which of the following statements best describes your situation regarding the residence we addressed our letter of invitation to?

- ○ owner not occupying
- ○ owner occupying year-round
- ○ owner occupying more than half the year
- ○ owner occupying less than half the year
- ○ tenant occupying year-round
- ○ tenant occupying more than half the year
- ○ tenant occupying less than half the year
- ○ occupant without payment of rent

**Wildfire**

*Q4.* What is the dominant land cover at your residence?

- ○ spruce forest
- ○ birch or aspen forest
- ○ high shrub more than 5ft tall
- ○ mixed forest
- ○ urban, mostly pavement, lawn, few trees
- ○ low shrub less than 5ft tall

*Q5.* How have you been affected by wildfire? (Select all that apply)

- ☐ had to stay indoors due to smoke
- ☐ had to reduce outdoor activities
- ☐ loss of income
- ☐ property loss
- ☐ external building loss
- ☐ lost home
- ☐ loss of pets and animals
- ☐ service outage (electricity, Internet, etc.)
- ☐ ____________ other (specify below)

*Q6.* What services were interrupted and for how long?

| | a day or less | several days but less than a week | a week and more |
|---|---|---|---|
| electricity | ○ | ○ | ○ |
| telephone, Internet | ○ | ○ | ○ |
| other (specify below) ____________ | ○ | ○ | ○ |
| other (specify below) ____________ | ○ | ○ | ○ |

*Q7.* How have service interruptions affected your household?

- ○ had to purchase a generator
- ○ freezer foods spoiled
- ○ other (specify below)

*Q8.* How effective are government agencies in responding to wildfire in your community?

- ○ very effective
- ○ somewhat effective

- ○ neutral
- ○ somewhat ineffective
- ○ very ineffective

*Q9.* How well prepared are government agencies for responding to wildfire in your community?

- ○ very prepared
- ○ somewhat prepared
- ○ neutral
- ○ somewhat not prepared
- ○ not very prepared

*Q10.* What level of responsibility do private landowners have in preparing for wildfire? (Select all that apply)

- ☐ participate in wildfire protection programs
- ☐ prepare their home and landscape
- ☐ participate in wildfire mitigation, neighborhood, or community council efforts
- ☐ prepare a written wildfire plan
- ☐ ____________ other (specify below)

*Q11.*

Which of the following wildfire mitigation activities were completed **on the house of your residence** over the past 5 years? (Select all that apply)

- ☐ installed fire resistant siding
- ☐ installed fire resistant roofing
- ☐ installed gutter guards
- ☐ installed screening over vents
- ☐ installed a chimney spark arrester
- ☐ cleaned roof and gutters
- ☐ closed eaves with horizontal soffit
- ☐ ____________ other (specify below)
- ☐ none

*Q12.*
Which of the following wildfire mitigation activities were completed **on the land parcel of your residence** over the past 5 years? (Select all that apply)

- ☐ removed all conifers within 15ft of house
- ☐ removed dead trees
- ☐ thinned conifers between 15 and 100ft from the house
- ☐ removed all dry and dead vegetation
- ☐ removed shrubs beneath trees
- ☐ pruned limbs of mature conifers
- ☐ thinned black spruce
- ☐ kept lawn at less than 3" and watered
- ☐ kept trees healthy and watered
- ☐ kept areas under stairs and decks free of debris
- ☐ provided 15ft of clearance between chimney and trees
- ☐ installed a 3ft permieter around home with non-burnables
- ☐ moved firewood at least 30ft away from house
- ☐ kept external fire-sprinkler system on property (>1000gal tank, pump, hoses)
- ☐ planted fire-resistant vegetation such as larch
- ☐ ____________ other (specify below)
- ☐ none

*Q13.* How much effort have members of your household put towards reducing wildfire hazards on your residence on average per year over the past 5 years?

persons on average per year ____________

hours on average per year ____________

*Q14.* How did you learn about wildfire mitigation activities? (Select all that apply)

- ☐ From the owner of my residence
- ☐ Alaska Firewise / Canada Firesmart
- ☐ Friends
- ☐ Neighbors
- ☐ Community Council Meetings

- [ ] Facebook/Social Media
- [ ] Community Meetings/Events
- [ ] Websites
- [ ] Radio
- [ ] TV
- [ ] ________ Other (specify below)

*Q15.* Do you support electric utilities shutting off power proactively during high fire danger, especially with high wind events?

- ○ definitely yes
- ○ probably yes
- ○ might or might not
- ○ probably not
- ○ definitely not

*Q16.* Have you participated in the following programs that prepare you for wildfire?

- ○ Alaska's Firewise
- ○ Canada's Firesmart
- ○ No

*Q17.* Have you completed the Alaska's Firewise or Canada's Firesmart program?

- ○ yes
- ○ no

*Q18.* Have you received a reimbursement from Alaska's Firewise program?

- ○ ________ yes, (Specify approximate amount below)
- ○ no
- ○ not sure

*Q19.* How has the wildfire hazard changed over the past ten years in the area surrounding your residence?

- ○ a lot more wildfire hazard
- ○ slightly more wildfire hazard
- ○ no change
- ○ slightly less wildfire hazard
- ○ a lot less wildfire hazard

*Q20.*

On a scale of 0 to 10 with 10 being "high risk" and 0 being "low risk" what level of risk do you feel wildfire presents to your property?

| | Low risk, no wildfire hazard | | | | | | | | | | High risk, must deal with hazard a lot | |
|---|---|---|---|---|---|---|---|---|---|---|---|---|
| | 0 | 1 | 2 | 3 | 4 | 5 | 6 | 7 | 8 | 9 | 10 | |
| wildfire | | | | | | | | | | | | ☐ |

**Ice**

*Q21.* In this section we will ask you about your experience with **ice hazards**. Ice hazards can be caused by rain-in-winter or by freeze-thaw. Rain-in-winter is rain falling on frozen ground, freezing on the surface and turning into ice. Freeze-thaw occurs when temperatures are above freezing melting snow or ice followed by temperatures below freezing. In this case, meltwater turns into black ice.

Note, we are **not** asking about heavy snow and winter storms in general. We **only** want to know about **ice**.

*Q22.* During freeze-thaw or rain-in-winter, does your driveway become hazardous?

- ○ yes
- ○ no

*Q23.* Is the road leading up to your property difficult to drive in icy conditions?

- ○ not at all, very easy, safe
- ○ slightly difficult
- ○ moderately difficult
- ○ considerably difficult/unsafe
- ○ extremely difficult/unsafe

*Q24.* How have you been affected by **ice** hazards? (Select all that apply)

- ☐ Car or other accident
- ☐ Service outage from ice-covered lines (electricity, Internet, etc.)
- ☐ Lost or damaged property
- ☐ Injury
- ☐ Permanent disability
- ☐ Missed work
- ☐ School or day-care closures
- ☐ Kids missed school/day care
- ☐ Left work early or arrived late
- ☐ Unable to participate in planned activities (shopping, medical care, going out to dinner, movies, etc)
- ☐ __________ Other (specify below)
- ☐ None

*Q25.* How much have you spent on damages or injury due to **ice** hazards in the past 2 years? (A guess is fine)

Expenses reimbursed by insurance __________

Expenses you paid not covered by insurance __________

*Q26.* What actions have you taken to mitigate **ice** hazards?

- ☐ Spread sand or gravel
- ☐ Spread ice melt
- ☐ Purchased winter tires/studded tires/tire chains
- ☐ Removed hard packed snow
- ☐ Purchased slip-resistant shoes/yaktrax/ice cleats

- ☐ Purchases studded snow tires
- ☐ ________ Other (specify below)
- ☐ None

*Q27.* In the past 2 years, what were your expenses for ice hazard mitigation? (A guess is fine)

Total expenses ________

Out-of-pocket expenses in case of insurance ________

*Q28.* In winter, what tires are on your vehicle?

- ○ all-season tires
- ○ summer tires
- ○ winter tires without studs
- ○ winter tires with studs

*Q29.* What services were interrupted and for how long due to **ice** covering powerlines?

| | a day or less | several days but less than a week | a week and more |
|---|---|---|---|
| electricity | ○ | ○ | ○ |
| telephone, Internet | ○ | ○ | ○ |
| other (specify below) ________ | ○ | ○ | ○ |

*Q30.* How have service interruptions affected your household?

- ○ Had to purchase a generator
- ○ Freezer foods spoiled
- ○ Other (specify below)

________

*Q31.* How has the **ice** hazard changed over the past ten years?

- a lot more ice hazard
- slightly more ice hazard
- no change
- slightly less ice hazard
- a lot less ice hazard
- don't know

*Q32.*

On a scale of 0 to 10 with 10 being "high risk" and 0 being "low risk" what level of risk do you feel **ice** hazards present to you?

| | Low risk, no ice hazard | | | | | High risk, must deal with hazard a lot | | | | | | |
|---|---|---|---|---|---|---|---|---|---|---|---|---|
| | 0 | 1 | 2 | 3 | 4 | 5 | 6 | 7 | 8 | 9 | 10 | |
| ice hazard | | | | | | | | | | | | |

**Permafrost Thaw**

*Q33.* In this section, we will ask you about permafrost thaw. Permafrost thaw can be slow with subtle changes, or rapid resulting in catastrophic ground collapse and opening up of sink holes.

*Q34.* Since you have been living at your current residence, have you seen ground subsidence or sink holes?

- yes
- no
- not sure

*Q35.* Do you believe the subsidence or sink holes are related to permafrost?

- not likely
- somewhat likely
- very likely

*Q36.* Are you aware of permafrost on the property were you reside or next to that property?

- ○ yes
- ○ no

*Q37.* Are you concerned about permafrost possibly being on the property where you reside?

- ○ yes
- ○ maybe
- ○ no

*Q38.* How has permafrost thaw affected the property where you reside? (Select all that apply)

- ☐ It is affecting utility access
- ☐ It is affecting road access
- ☐ Changed the vegetation
- ☐ Made the property unsafe
- ☐ It is affecting a septic tank
- ☐ It is affecting a foundation
- ☐ It is affecting a lawn and landscaping
- ☐ There is ground surface collapse
- ☐ It forms pools of standing water and erosion gullies
- ☐ Decreased my ability to sell the property
- ☐ Decreased property value
- ☐ [ ] Other (specify below)
- ☐ None

*Q39.* What prevention was done on the property where you reside to maintain frozen ground? (Select all that apply)

- ☐ built an elevated structure (on piling or post and pad)
- ☐ planted shrubs for shading around the perimeter of buildings

- ☐ dug out frozen soil and backfilled with gravel
- ☐ installed gutters to route water away from foundations
- ☐ installed an above ground septic system
- ☐ built up a gravel pad
- ☐ built extended eaves
- ☐ built in an adjustable foundation
- ☐ installed active cooling systems (refrigeration/heat pumps)
- ☐ ________ other (specify below)
- ☐ none

*Q40.* How has the permafrost hazard in the surrounding area of your residence changed over the past ten years?

- ○ a lot more permafrost thaw
- ○ slightly more permafrost thaw
- ○ no change
- ○ slightly less permafrost thaw
- ○ a lot less permafrost thaw

*Q41.*

On a scale of 0 to 10 with 10 being "high risk" and 0 being "low risk" what level of risk do you feel permafrost thaw presents to the property where you reside?

| | Low risk, no permafrost nearby | | | | | | | | | | High risk, must deal with hazard a lot | |
|---|---|---|---|---|---|---|---|---|---|---|---|---|
| | 0 | 1 | 2 | 3 | 4 | 5 | 6 | 7 | 8 | 9 | 10 | |
| permafrost thaw | | | | | | | | | | | | ☐ |

**Insurance MitigationCost**

*Q42.* Does your homeowners insurance policy cover losses due to wildfire?

- ○ yes
- ○ pretty sure

- not sure
- no

*Q43.* Why do you not have homeowners insurance that covers wildfire? (Select all that apply)

- not worth the cost
- doesn't cover personal items (tools, etc.)
- property not eligible
- didn't think I would need it
- doesn't cover outbuildings (sheds, etc.)
- too expensive
- ____________ other (specify below)

*Q44.* Which insurance company provides your homeowners policy?

- AllState
- Country Financial
- Geico
- Liberty Mutual
- State Farm
- Horace Mann
- Alaska Pacific Insurance
- Conrad-Houston Insurance
- USAA
- Umialik
- ____________ Other (specify below)
- I prefer not to specify!

*Q45.* Did your insurance company encourage or require you to prepare the property for wildfire?

- they required it to cover the property
- no

- ○ [ ] other (specify below)

*Q46.* How much have you spent on wildfire mitigation over the past 5 years? (A guess is fine)

Expenses reimbursed by insurance [ ]

Expenses you paid not covered by insurance [ ]

*Q47.* Does your homeowners insurance cover damages from permafrost?

- ○ yes
- ○ no
- ○ don't know

*Q48.* How much have you spent on permafrost mitigation since owning your residence? (A guess is fine)

Expenses reimbursed by insurance [ ]

Expenses you paid not covered by insurance [ ]

**Socio-demographics**

*Q49.* How many years have you lived at your residence?

- ○ 0 to 10
- ○ 11 to 20
- ○ 21 to 30
- ○ more than 30

*Q50.* What is your age?

- ○ between 18 and 30
- ○ between 31 and 40
- ○ between 41 and 50
- ○ between 51 and 60
- ○ between 61 and 70
- ○ between 70 and 80

- older than 80

*Q51.* How many people are living in your household currently?

persons ______

*Q52.* Do you have children under 18 living in your household?

- yes
- no

*Q53.* Which category best describes your 2020 household income before taxes?

- Less than $25,000
- $25,001 - $50,000
- $50,001 - $75,000
- $75,001 - $100,000
- $100,001 - $125,000
- $125,001 - $150,000
- $150,001 - $200,000
- $200,001 - $250,000
- More than $250,000

*Q54.* For future follow-up, please select all that apply. This is voluntary.

- I would like to receive the research results from this study.
- I am willing to participate in follow-up research on climate change related topics.
- I am willing to have permafrost monitoring instrumentation be installed on my property.

*Q55.* Please enter your email address, below.

email ______

Powered by Qualtrics
