# Supplementary material for: Increasing multi-hazard climate risk and financial and health impacts on northern homeowners
Source: Ambio. 2023 Nov 13;53(3):389–405. doi: 10.1007/s13280-023-01951-z (PMC10837396; doi:10.1007/s13280-023-01951-z)
Supplement: Supplementary file 2 — Supplementary file2 (PDF 93 KB) [file 13280_2023_1951_MOESM2_ESM.pdf]

Ambio

Supplementary Information 2

Increasing multi-hazard climate risk and financial and health impacts on northern homeowners

Tobias Schwoerer a*, Jennifer I. Schmidt b, Matthew Berman b, Peter Bienik a, Louise M. Farquharson c Dmitry Nicolsky c, James Powell d, Rachel Roberts b, Rick Thoman a, Robert Ziel c

a International Arctic Research Center, University of Alaska Fairbanks, PO Box 75734, Fairbanks, AK 99775-7340, USA,
b Institute of Social and Economic Research, University of Alaska Anchorage, 3211 Providence Dr. Anchorage, AK 99509, USA
c Geophysical Institute, University of Alaska Fairbanks, PO Box 757320, Fairbanks, AK, 99775-7340, USA
d Alaska Coastal Rainforest Center, University of Alaska Southeast, 21066 Auke Lake Way, Juneau, AK 99801, USA

* corresponding author: tschwoerer@alaska.edu, +1 (907) 786 5404

Code file in R Studio Rmarkdown

```
---
title: "AURA"
author: "Tobias Schwoerer"
date: "5/11/2023"
output: html_document
---

# Data import
We suggest uploading all data from the Arctic Data Center (ADC) into one folder
where this code file is also located.
Below is code to be used in an Rmd, R Markdown file. Please go to R Project and
install free of charge open source software to open this file,
best in conjunction with R Studio.

```{r}
setwd(dirname(rstudioapi::getActiveDocumentContext()$path))

library(dplyr)
library(ggplot2)
library(tidyr)
library(EnvStats)
library(magrittr)
library(survey)
library(stringi)
```
```

```
library(stringr)
library(PerformanceAnalytics)
library(janitor)
library(svyweight)
library(MatchIt)
library(viridis)
library(hrbrthemes)
library(cowplot)

#probability sample
dataP <-
read.csv("https://arcticdata.io/metacat/d1/mn/v2/object/urn%3Auuid%3A887bf14d-e27e-
437a-a59f-1aa8d9740ed9",stringsAsFactors = F)
dataP <- dataP[-c(1,2),] #deleting left over rows from Qualtrics
row.names(dataP) <- NULL # resetting rownumbers

anc <-
read.csv("https://arcticdata.io/metacat/d1/mn/v2/object/urn%3Auuid%3Acc433a89-eeec-
4cf7-860a-37d640f613f9", stringsAsFactors = F)
fai <-
read.csv("https://arcticdata.io/metacat/d1/mn/v2/object/urn%3Auuid%3A0ac3965a-f742-
40ec-955c-9c8b34c128d1", stringsAsFactors = F)

#revised wildfire hazard categories and metrics
newFCanc <-
read.csv("https://arcticdata.io/metacat/d1/mn/v2/object/urn%3Auuid%3A3de1744f-eb5d-
44d2-a701-2a30c2239fb9",stringsAsFactors = F)
newFCfai <-
read.csv("https://arcticdata.io/metacat/d1/mn/v2/object/urn%3Auuid%3A20c22588-77a9-
463c-b64a-8e76e251a1d4",stringsAsFactors = F)

#revised permafrost category for Anchorage
newPermCanc <-
read.csv("https://arcticdata.io/metacat/d1/mn/v2/object/urn%3Auuid%3Ad2b5388b-494a-
4db0-addd-bf90efa02340",stringsAsFactors = F)

#strata for comparing sample and population proportions, etc.
p_selection <-
read.csv("https://arcticdata.io/metacat/d1/mn/v2/object/urn%3Auuid%3A77339f0c-bcfc-
470c-a4a7-488a1c76c9cc",fileEncoding = 'UTF-8-BOM')
strat_Hcat2 <-
read.csv("https://arcticdata.io/metacat/d1/mn/v2/object/urn%3Auuid%3A9268f4b9-f7e6-
4f32-a539-c96d8d161438",fileEncoding = 'UTF-8-BOM')

#parcels (assuming = households) by stratum
anc_parcels <-
read.csv("https://arcticdata.io/metacat/d1/mn/v2/object/urn%3Auuid%3A8a3a8e8a-d2f9-
4923-8f9f-c684de3fd16a")
fai_parcels <-
read.csv("https://arcticdata.io/metacat/d1/mn/v2/object/urn%3Auuid%3A613f7977-d7ce-
```

~~~
4fa1-9d98-c404ecd38161")

#public use microdata from the Census
pumsX <-
read.csv("https://arcticdata.io/metacat/d1/mn/v2/object/urn%3Auuid%3A0ac97c0b-756e-
4741-8706-eacc276f29e3",stringsAsFactors = F)
```

#Generating a data file for analysis
Here we add other reference tables with information from the hazard assessment and
property data bases
```{r}
#generating a reference table for adding strata ID, ice and permafrost hazard
categories, property values, etc..
#reference table for Anchorage
anc1 <- anc%>%
  select(Email,Strat, Parcel_ID, Lot_Size, Appraise_2, Land_Value, Rain_Cat)%>%
  rename(TotalValue=Appraise_2,
         LandValue=Land_Value,
         LotSize=Lot_Size)
anc1 <- anc1%>%
  left_join(newFCanc, by="Parcel_ID")%>%
  rename(FireM=Fire5mMean,
         Fire=FireCatJS,
         Ice=Rain_Cat)
#joining revised permafrost category for Anchorage
anc1 <- anc1%>%
  left_join(newPermCanc, by="Parcel_ID")%>%
  rename(Perm=PermCat2)%>%

select("Email","Strat","Fire","Ice","Perm","FireM","LandValue","TotalValue","LotSiz
e","Parcel_ID")

#reference table for Fairbanks
fai1 <- fai%>%
  select(Email, PAN, Rain_Cat_NB, Perm_Cat_2, Strat,Land_Value_1, Total_Valu_1)%>%
  rename(Ice = Rain_Cat_NB,
         Perm = Perm_Cat_2,
         LandValue = Land_Value_1,
        TotalValue = Total_Valu_1)
fai1['LotSize'] <- NA
fai1 <- fai1 %>%
  left_join(newFCfai, by="PAN")%>%

select("Email","Strat","FireCatJS","Ice","Perm","Fire5mMean","LandValue","TotalValu
e","LotSize","PAN")%>%
  rename(FireM=Fire5mMean,
         Fire=FireCatJS,
         Parcel_ID=PAN)
~~~

````
refs <- rbind(fai1, anc1)

# using consistent column header for Email
dataP <- dataP%>%
  rename(Email=RecipientEmail)
dataP$City <- substr(dataP$Email,1,3)

#adding reference information to the data
data <- dataP%>%
  left_join(refs, by="Email")
```

#Table 1
survey response and sample characteristics
```{r}
#comparing sample to population
strat_response <- data%>%
  group_by(City,Strat)%>%
  summarise(n_count=n())

refs2 <- refs
refs2$City <- substr(refs2$Email,1,3)
strat_pop <- refs2%>%
  group_by(City,Strat)%>%
  summarise(N_count=n())

#creating a reference table for strata
seqAnc <- data.frame(Strat = seq(1:27))%>%
  mutate(City="Anc")
seqFai <- data.frame(Strat = seq(1:27))%>%
  mutate(City="Fai")
strat_base = rbind(seqAnc, seqFai)

selection_overview <- strat_base%>%
  left_join(strat_pop,by=c("City"="City", "Strat"="Strat"))%>%
  left_join(strat_response, by=c("City"="City", "Strat"="Strat"))%>%
  left_join(p_selection, by=c("City"="City", "Strat"="Strat"))

selection_overview$p <- with(selection_overview,
ifelse(City=="Anc",round(n_count/320,digits=4),ifelse(City=="Fai",round(n_count/379
,digits=4),round(n_count/53,digits=4))))
selection_overview$prob <- round(selection_overview$prob,digits=4)
selection_overview <- selection_overview%>%
  left_join(strat_Hcat2,by="Strat")

#Table A1
sample_char <- selection_overview%>%
  group_by(City,Fire,Ice,Perm)%>%
  summarise(FreqN = sum(N_count),
            Freqn = sum(n_count))
````

```
#write.csv(sample_char, "Table_A1.csv")

age_char <- data%>%
  group_by(City,Q50)%>%
  summarise(n_count=n())
#write.csv(age_char, "age.csv")

#response counts by city
res_by_city <- strat_response%>%
  group_by(City)%>%
  summarise(n_count=sum(n_count))

#Q47 insurance covering permafrost issues
data$Q47 <- as.numeric(data$Q47)
data$PermInsure <- with(data, ifelse(is.na(Q47),NA,ifelse(Q47==1,1,0)))

#Q49 years lived at residence
longevity <- data%>%
  group_by(City,Q49)%>%
  summarise(count=n())
#write.csv(longevity, "longevity.csv")
#Q49 years lived in city, using midpoint of intervals shown in the survey
instrument
data$Q49n <- recode(data$Q49, #the variable you want to recode, the old is in ` `
the new on the right hand side
                           `1` = 5,
                           `2` = 15,
                           `3` = 25,
                           `4` = 35)



#Q50 age, using midpoints of the intervals shown in the survey instrument
data$Q50 <- as.numeric(data$Q50)
data$Q50n <- recode(data$Q50, #the variable you want to recode, the old is in ` `
the new on the right hand side
                           `1` = 24,
                           `2` = 35,
                           `3` = 45,
                           `4` = 55,
                           `5` = 65,
                           `6` = 75,
                           `7` = 85)
#income recoding
data$IncNew <- recode(data$Q53, #the variable you want to recode, the old is in ` `
the new on the right hand side
                           `1` = 25000,
                           `2` = 37500,
                           `3` = 62500,
                           `4` = 87500,
```

```
                           `5` = 112500,
                           `6` = 137500,
                           `7` = 175000,
                           `8` = 225000,
                          `9` = 250000)

#response property owner versus tenant
data$Q3 <- as.numeric(data$Q3)
data$Q3owner <- with(data,ifelse(Q3==8|Q3==1|Q3==4|Q3==5,1,0))

#household size
data$Q51_1 <- ifelse(data$Q51_1=="",NA,data$Q51_1)
data$Q51_1 <- as.numeric(data$Q51_1)
#Children under 18 living in hh
data$Q52 <- as.numeric(data$Q52)
data$Q52n <- recode(data$Q52, #the variable you want to recode, the old is in ` ` the new on the right hand side
                           `1` = 1,
                           `2` = 0)

#Table 1 sample characteristics
ch <- data%>%
  group_by(City)%>%
  summarize(meanInc = mean(IncNew, na.rm=T),
            sdInc = sd(IncNew, na.rm=T),
            meanAge = mean(Q50n, na.rm=T),
            sdAge = sd(Q50n, na.rm=T),
            meanLong = mean(Q49n, na.rm=T),
            sdLong = sd(Q49n, na.rm=T),
            meanRenter = mean(Q3owner, na.rm=T),
            sdRenter = sd(Q3owner, na.rm=T),
            meanHHsize = mean(Q51_1, na.rm=T),
            sdHHsize = sd(Q51_1, na.rm=T),
            meanChild = mean(Q52n, na.rm=T),
            sdChild = sd(Q52, na.rm=T))

 ch <- data%>%
 group_by(City) %>%
  summarise(quantile = scales::percent(c(0.25, 0.5, 0.75), na.rm=T),
            Inc = quantile(IncNew, c(0.25, 0.5, 0.75), na.rm=T),
            Age = quantile(Q50n, c(0.25, 0.5, 0.75), na.rm=T),
            Longevity = quantile(Q49n, c(0.25, 0.5, 0.75), na.rm=T),
            hhSize = quantile(Q51_1, c(0.25, 0.5, 0.75), na.rm=T)
            )
```

#Data cleaning and preparation

Note, to prepare data for calculating affected number of households by hazard, we cannot rely on answers to Q2, need to use impact specific answers: for fire Q5, for ice use Q24 and for permafrost use Q38

```{r}
data$Q2 <- as.numeric(data$Q2)
#creating variable for proportion of population affected
data$FireAffectedPop <- with(data, ifelse(Q5=="",0,1))
data$IceAffectedPop <- with(data, ifelse(Q24==""|Q24=="5",0,1))
data$PermAffectedPop <- with(data, ifelse(Q38==""|Q38=="6",0,1))
#with NA values for those who did not respond
data$FireAffected <- with(data, ifelse(Q5=="",NA,1))
data$IceAffected <- with(data, ifelse(Q24=="",NA,ifelse(Q24=="5",0,1)))
data$PermAffected <- with(data, ifelse(Q38=="",NA,ifelse(Q38=="6",0,1)))

#making sure variables are numeric
data$Q20_1 <- as.numeric(data$Q20_1) #fire risk rating
data$Q32_1 <- as.numeric(data$Q32_1) #ice risk rating
data$Q41_1 <- as.numeric(data$Q41_1) #permafrost risk rating

#Fire Mitigation Effort
#data cleaning fire mitigation person hours
dirty <- c("","Medium","? hard to say","All summet","low","?","a
little","Limited","n/a","100+","100","0.01","100%","80%","50%","56%","75%","20
hours")
data$Q13_1 <- with(data,ifelse(Q13_1%in% dirty,NA,Q13_1))
data$Q13_1 <- with(data,ifelse(Q13_1=="None",0,ifelse(Q13_1=="",NA,ifelse(Q13_1=="1
plus help from neighbors",2,ifelse(Q13_1=="one"|Q13_1=="One",1,Q13_1)))))
data$Q13_1 <- as.numeric(data$Q13_1)
#cleaning fire mitigation hours per year
dirty2 <- c("many","? Not many","All Summer","? A
lot","10-Jun","?????","some","good")
data$Q13_2 <- with(data,ifelse(Q13_2%in% dirty2,NA,Q13_2))
data$Q13_2 <- with(data,ifelse(Q13_2=="None",0,ifelse(Q13_2=="",NA,ifelse(Q13_2=="?
80 - 100? I donâ€™t keep a
record",90,ifelse((Q13_2=="Unknown"|Q13_2=="unknown"|Q13_2=="don't
know"|Q13_2=="n/a"),NA,ifelse(Q13_2=="20-30+",30,ifelse(Q13_2=="20-30",25,ifelse(Q1
3_2=="50-60",55,ifelse((Q13_2=="10-20"|Q13_2=="10 to
20"),15,ifelse(Q13_2=="0.1",0,ifelse(Q13_2=="30-40",35,Q13_2))))))))))

#cleaning remaining strings and whitespace in the hours
data$Q13_2n <- readr::parse_number(data$Q13_2, na = c("", "NA","All summer"),
trim_ws = TRUE)

#calculating effort in person hours
data$FireEffort <- with(data,Q13_2n*Q13_1, na.rm=T)
#removing 9 outliers that > 1000 pers hours
data$FireEffort <- with(data,ifelse(FireEffort>1000,NA,FireEffort))


#cleaning remaining strings and whitespace in total expense on ice haz mitigation
data$Q27_1 <- as.character(data$Q27_1)
data$Q27_1 <- readr::parse_number(data$Q27_1, na = c("","unknown",
"???","NA","?","None","Mainly my own work. When excessive ice commercial gravel
```

```
dump $100","New tires and ice melt","o","O"), trim_ws = TRUE)
data$Q27_2 <- as.character(data$Q27_2)
data$Q27_2 <- readr::parse_number(data$Q27_2, na = c("","$",
"?","n/a","--",",","None","NA","o","O","/"), trim_ws = TRUE)

#Injury reimbursed by insurance Q25_1 and not covered Q25_2
data$Q25_1 <- as.numeric(data$Q25_1)
data$Q25_2 <- as.numeric(data$Q25_2)

#cleaning
data$Q46_1 <- readr::parse_number(data$Q46_1, na = c(""), trim_ws = TRUE)
data$Q46_2 <- readr::parse_number(data$Q46_2, na = c(""), trim_ws = TRUE)
data$Q46_1 <- as.numeric(data$Q46_1)
data$Q46_2 <- as.numeric(data$Q46_2)
#outliers in fire mitigation
data$Q46_2 <- ifelse(data$Q46_2==25000,NA,data$Q46_2)
data$Q46_1 <- ifelse(data$Q46_1==25000,NA,data$Q46_1)
#making sure that missing values of either one of the fire mitigation cost
questions are coded as 0 to not be missed in the total sum of mitigation costs
data$Q46_2 <- ifelse(is.na(data$Q46_2)&!is.na(data$Q46_1),0,data$Q46_2)
data$Q46_1 <- ifelse(is.na(data$Q46_1)&!is.na(data$Q46_2),0,data$Q46_1)
data$FireMitCost <- data$Q46_1+ data$Q46_2
firewise <- data%>%
  filter(Q16==1)%>%
  group_by(City)%>%
  summarise(count=n())

#permafrost related cleaning
data$Q48_1 <- readr::parse_number(data$Q48_1, na = c(""), trim_ws = TRUE)
data$Q48_2 <- readr::parse_number(data$Q48_2, na = c(""), trim_ws = TRUE)
data$Q48_1 <- as.numeric(data$Q48_1)
data$Q48_2 <- as.numeric(data$Q48_2)
#setting outliers to NA
data$Q48_2 <- ifelse(data$Q48_2==250000,NA,data$Q48_2)
data$Q48_1 <- ifelse(data$Q48_1==250000,NA,data$Q48_1)

#making sure that missing values of either one of the fire mitigation cost
questions are coded as 0 to not be missed in the total sum of mitigation costs
data$Q48_2 <- ifelse(is.na(data$Q48_2)&!is.na(data$Q48_1),0,data$Q48_2)
data$Q48_1 <- ifelse(is.na(data$Q48_1)&!is.na(data$Q48_2),0,data$Q48_1)
data$PFMitCost <- data$Q48_1 + data$Q48_2

hh_w_pf_cost <- data%>%
  filter(Q48_2>0)%>%
  group_by(City)%>%
  summarise(count=n())

#recoding Q8 and 9, government response to wildfire
data$Q8 <- as.numeric(data$Q8) #effective gov response
data$Q9 <- as.numeric(data$Q9) #gov preparation
```

```
data$Q8n <-
with(data,ifelse(Q8==1,2,ifelse(Q8==2,1,ifelse(Q8==3,0,ifelse(Q8==4,-1,-2)))))
data$Q9n <-
with(data,ifelse(Q9==1,2,ifelse(Q9==2,1,ifelse(Q9==3,0,ifelse(Q9==4,-1,-2)))))

#support for shutting off power during high fire danger and wind
data$Q15 <- as.numeric(data$Q15)
#participation in Firewise program
data$Q16 <- as.numeric(data$Q16)
data$Firewise <- with(data, ifelse(Q16==1,1,0))

#preparing dataset for correlation analysis
data$Q49 <- as.numeric(data$Q49)
data$Q50 <- as.numeric(data$Q50)
data$Q53 <- with(data, ifelse(Q53=="",NA,ifelse(Q53=="5,6",6,
ifelse(Q53=="6,7",7,ifelse(Q53=="3,4",4,ifelse(Q53=="4,6",5,Q53))))))
data$Q53 <- as.numeric(data$Q53)
data$Q42 <- as.numeric(data$Q42)
data$Q42n <- recode(data$Q42, #the variable you want to recode, the old is in ` `
the new on the right hand side
                           `1` = 1,
                           `2` = 1,
                            `3`= 0,
                            `4`=0)

#ice
data$drway <- as.numeric(data$Q23)
data$Q32_1 <- as.numeric(data$Q32_1)
#preparing to plot correlation figure
data$Q28 <- as.numeric(data$Q28)
data$tires <- data$Q28
data$Q25_1 <- as.numeric(data$Q25_1)
data$Q25_2 <- as.numeric(data$Q25_2)
data$InjTotCost <- data$Q25_1 + data$Q25_2

#recoding land cover
data$Q4 <- as.numeric(data$Q4)
#data$Q4 <- recode(data$Q4, '1'= 5, '2'= 3,'3'= 2,'4'= 4,'5'= 1,'6'= 2)

data$Q41_ <- as.numeric(data$Q41_1)
data$Q34 <- as.numeric(data$Q34)
data$Q34n <- ifelse(data$Q34==1,1,0)
data$Q35 <- as.numeric(data$Q35)
data$Q35 <- ifelse(data$Q35==1,0,1)
data$subsid <- data$Q34n*data$Q35  #respondent saw sinkholes on property and
believes these are related to permafrost
data$Q36 <- as.numeric(data$Q36)
data$Q37 <- as.numeric(data$Q37)
data$aware <- ifelse(data$Q36==1,1,0)
```

~~~~
data$concern <- ifelse(data$Q37==1,2,ifelse(data$Q37==2,1,0))  #left this out of
the corr figure as there are too many NAs
```

#Survey data analysis
##Preparing PUMS data for income variable
```{r}
pumsX$HINCP <- as.numeric(pumsX$HINCP)
pumsX$Inc <- with(pumsX, ifelse(HINCP==-60000,NA,HINCP))
pumsX$Inc <- as.numeric(pumsX$Inc)
pumsX$Inc <- with(pumsX, ifelse(HINCP<=0,1,HINCP))
pumsX$IncCat <-
cut(pumsX$Inc,breaks=c(0,25000,50000,75000,100000,125000,150000,200000,250000,80000
0),labels=c("1","2","3","4","5","6","7","8","9"))
ANCpumsInc<- pumsX%>%
  filter(PUMA<300)%>%
  group_by(IncCat)%>%
  summarise(Freq=sum(WGTP))%>%
  rename(Q53n=IncCat)
#Anchorage total number of parcels in sampling frame, then adjusting margin table
accordingly
ancParcels <- 82148
ANCpumsInc2 <- ANCpumsInc%>%
  mutate(total=sum(Freq),
         Prop=Freq/total)
ANCpumsInc2$Freq <- round(ANCpumsInc2$Prop*ancParcels,digits=0)
ANCpumsInc2 <- ANCpumsInc2%>%select(-c("Prop","total"))

FAIpumsInc<- pumsX%>%
  filter(PUMA==300)%>%
  group_by(IncCat)%>%
  summarise(Freq=sum(WGTP))%>%
  rename(Q53n=IncCat)

#Fairbanks total number of parcels in sampling frame, then adjusting margin table
accordingly
faiParcels <- 18074
FAIpumsInc2 <- FAIpumsInc%>%
  mutate(total=sum(Freq),
         Prop=Freq/total)
FAIpumsInc2$Freq <- round(FAIpumsInc2$Prop*faiParcels,digits=0)
FAIpumsInc2 <- FAIpumsInc2%>%select(-c("Prop","total"))

#For the income variable, setting 88 NA values to the median hh income category for
Anchorage and Fairbanks
data$Q53n <-
with(data,ifelse(is.na(Q53)&City=="Fai",3,ifelse(is.na(Q53)&City=="Anc",4,Q53)))
```

##Preparing data for survey analysis
~~~~

Below we generate A) sample margins using the Public Use Microdata Sample (PUMS) from the U.S. Census. The primary sampling unit (PSU) was the household. Household size (Q51_1) was used as the household weighting variable (source: PUMS ACS 2021 one year estimates). Then we added the above to each dataset for survey analysis, splitting data into an Anchorage and Fairbanks dataset for further analysis.
The finite population correction was set to the probability of selection consistent with probability proportional to size (PPS) without replacement. Some useful reading: survey package example, #see
https://rstudio-pubs-static.s3.amazonaws.com/289413_af0aea8ae0f940fa8b65e14a36b72cac.html

````
```{r}
#preparing data for below survey analysis
#ICE
data$Q25_1 <- as.numeric(data$Q25_1)#ice hazard damages and injuries costs,
reimbursed by insurance
data$Q25_2 <- as.numeric(data$Q25_2)#ice hazard damages and injuries costs, out of
pocket
data$ice_injury <- with(data, ifelse(Q25_1>0|Q25_2>0,1,0))#creating variable
indicating households with injury or damages from ice hazard
data$Q27_1 <- as.numeric(data$Q27_1) #ice hazard mitigation costs over past 2
years, total
data$Q27_2 <- as.numeric(data$Q27_2) #ice hazard mitigation costs over past 2
years, out-of-pocket expense
data$IceMitCost <- data$Q27_1
data$Q31 <- as.numeric(data$Q31)
data$IceTrend <- with(data, ifelse(Q31==1|Q31==2,1,0))

#FIRE
data$Q19 <- as.numeric(data$Q19)#has fire hazard increased in past 10 years
data$FireTrend <- with(data, ifelse(Q19==1|Q19==2,1,0))
data$PwrShutOff <- with(data, ifelse(Q15==1|Q15==2,1,0))
data$Q46_1 <- as.numeric(data$Q46_1) #fire mitigation costs over past 5 years
data$Q46_2 <- as.numeric(data$Q46_2)

#PF
data$Q48_1 <- as.numeric(data$Q48_1) #permafrost mitigation costs since owning
residence
data$Q48_2 <- as.numeric(data$Q48_2)
data$Q40 <- as.numeric(data$Q40)
data$PermTrend <- with(data, ifelse(Q40==1|Q40==2,1,0))

#creating city specific datasets and adding probability of selection to specify the
final population correction
ancData <- data%>%
  subset(City=="Anc")%>%
  left_join(p_selection, by=c("Strat"="Strat","City"="City"))
ancData <- ancData%>%
  mutate(IncomeQuantile = cut(ancData$IncNew, unique(quantile(ancData$IncNew,
seq(0, 1, 0.25),na.rm=T)), labels = FALSE, na.rm=T ))
````
````

````
faiData <- data%>%
  subset(City=="Fai")%>%
  left_join(p_selection, by=c("Strat"="Strat","City"="City"))
faiData <- faiData%>%
  mutate(IncomeQuantile = cut(faiData$IncNew, unique(quantile(faiData$IncNew,
seq(0, 1, 0.25),na.rm=T)), labels = FALSE, na.rm=T))
```

##raking with income variable
Source: https://sdaza.com/blog/2012/raking/
Rule of thum, truncate weights that are greater than 5 times the mean of weights
```{r}
#Anchorage
#computing weights by first telling R about the stratified survey design and
population sizes by strata
options(survey.lonely.psu="adjust") #see
https://r-survey.r-forge.r-project.org/survey/exmample-lonely.html
ancDesign <- svydesign(id=~1,strata=~Strat, data=ancData, fpc=~prob)
weighted_ancData <- rake(design = ancDesign,
                         sample.margins = list(~Q53n),
                         population.margins = list(ANCpumsInc2) )
summary(weights(weighted_ancData))
#max weights are below 5*mean weight, so no trimming
w_ancData_trim <- weighted_ancData
#w_ancData_trim <- trimWeights(weighted_ancData, lower=50, upper=7400, strict=TRUE)
#trimming weights because some are too large
#summary(weights(w_ancData_trim))
svymean(~IncNew, w_ancData_trim, na.rm=T)
#Census 88,878

#Fairbanks
faiDesign <- svydesign(id=~1,strata=~Strat, data=faiData, fpc=~prob)
weighted_faiData <- rake(design = faiDesign,
                         sample.margins = list(~Q53n),
                         population.margins = list(FAIpumsInc2))
summary(weights(weighted_faiData))
w_faiData_trim <- weighted_faiData
#w_faiData_trim <- trimWeights(weighted_faiData, lower=25, upper=17000,
strict=TRUE) #trimming weights because some are too large
#summary(weights(w_faiData_trim))
svymean(~IncNew, w_faiData_trim, na.rm=T)
#Census 64,375
```

#Figure 2
using weighted survey data
income and hazard proportions
```{r}
a <- svyby(~Fire, ~IncomeQuantile, w_ancData_trim, svytotal, na.rm=T)
a <- a%>%
````

```
 round(digits=0)%>%
  as.data.frame()%>%
  pivot_longer(2:4,names_to = c("Fire"), values_to = "n")%>%
  select(c("IncomeQuantile","Fire","n"))
a$Fire <- str_sub(a$Fire,5)
a <- a%>%
  arrange(factor(Fire, levels = c("High","Medium","Low")))
a$Fire <- as.factor(a$Fire)
a$Fire <- ordered(a$Fire, levels = c("High","Medium","Low"))
a$City <- "Anc"

b <- svyby(~Fire, ~IncomeQuantile, w_faiData_trim, svytotal, na.rm=T)
b <- b%>%
 round(digits=0)%>%
  as.data.frame()%>%
  pivot_longer(2:4,names_to = c("Fire"), values_to = "n")%>%
  select(c("IncomeQuantile","Fire","n"))
b$Fire <- str_sub(b$Fire,5)
b <- b%>%
  arrange(factor(Fire, levels = c("High","Medium","Low")))
b$Fire <- as.factor(b$Fire)
b$Fire <- ordered(b$Fire, levels = c("High","Medium","Low"))
b$City <- "Fai"
quantFire <- rbind(a,b)

FirePlot <- ggplot(quantFire, aes(fill=Fire, x=IncomeQuantile, y=n)) +
    geom_bar(width = 0.6, position="fill", stat="identity") +
    theme_classic() +
     scale_fill_viridis(discrete=T,option = "E") +
    theme(axis.text = element_text(size = 7))  +
    facet_wrap(~City, labeller = labeller(City = c("Anc" = "Anchorage","Fai" =
"Fairbanks"))) +
    theme_ipsum() +
    theme(legend.position="none") +
    theme(axis.title = element_text(vjust = 0)) +
    xlab("Income quartile")+
  ylab("% in hazard zone") +
  scale_y_continuous(labels = scales::percent) +
  theme(axis.title.y = element_text(vjust=+2))
FirePlot
ggsave("Fig2-Fire.tif", plot = FirePlot, device = "tiff", path = NULL, scale = 1.0,
width = 140, height = 85, units = "mm",dpi = 300, limitsize = T)

#Ice hazard plot
c <- svyby(~Ice, ~IncomeQuantile, w_ancData_trim, svytotal, na.rm=T)
c <- c%>%
 round(digits=0)%>%
  as.data.frame()%>%
  pivot_longer(2:4,names_to = c("Ice"), values_to = "n")%>%
  select(c("IncomeQuantile","Ice","n"))
```

```
c$Ice <- str_sub(c$Ice,4)
c <- c%>%
  arrange(factor(Ice, levels = c("High","Medium","Low")))
c$Ice <- as.factor(c$Ice)
c$Ice <- ordered(c$Ice, levels = c("High","Medium","Low"))
c$City <- "Anc"

d <- svyby(~Ice, ~IncomeQuantile, w_faiData_trim, svytotal, na.rm=T)
d <- d%>%
 round(digits=0)%>%
  as.data.frame()%>%
  pivot_longer(2:4,names_to = c("Ice"), values_to = "n")%>%
  select(c("IncomeQuantile","Ice","n"))
d$Ice <- str_sub(d$Ice,4)
d <- d%>%
  arrange(factor(Ice, levels = c("High","Medium","Low")))
d$Ice <- as.factor(d$Ice)
d$Ice <- ordered(d$Ice, levels = c("High","Medium","Low"))
d$City <- "Fai"
quantIce <- rbind(c,d)

IcePlot <- ggplot(quantIce, aes(fill=Ice, x=IncomeQuantile, y=n)) +
    geom_bar(width = 0.6, position="fill", stat="identity") +
    theme_classic() +
     scale_fill_viridis(discrete=T,option = "E") +
    theme(axis.text = element_text(size = 7))  +
    facet_wrap(~City, labeller = labeller(City = c("Anc" = "Anchorage","Fai" =
"Fairbanks"))) +
    theme_ipsum() +
    theme(legend.position="none") +
    xlab("Income quartile")+
 ylab("% in hazard zone") +
  scale_y_continuous(labels = scales::percent)+
   theme(axis.title.y = element_text(vjust=+2))
IcePlot
ggsave("Fig2-Ice.tif", plot = IcePlot, device = "tiff", path = NULL, scale = 1.0,
width = 140, height = 85, units = "mm",dpi = 300, limitsize = T)

#Permafrost hazard plot
e <- svyby(~Perm, ~IncomeQuantile, w_ancData_trim, svytotal, na.rm=T)
e <- e%>%
 round(digits=0)%>%
  as.data.frame()%>%
  pivot_longer(2:4,names_to = c("Perm"), values_to = "n")%>%
  select(c("IncomeQuantile","Perm","n"))
e$Perm <- str_sub(e$Perm,5)
e <- e%>%
  arrange(factor(Perm, levels = c("High","Medium","Low")))
e$Perm <- as.factor(e$Perm)
e$Perm <- ordered(e$Perm, levels = c("High","Medium","Low"))
```

````
e$City <- "Anc"

f <- svyby(~Perm, ~IncomeQuantile, w_faiData_trim, svytotal, na.rm=T)
f <- f%>%
 round(digits=0)%>%
  as.data.frame()%>%
  pivot_longer(2:4,names_to = c("Perm"), values_to = "n")%>%
  select(c("IncomeQuantile","Perm","n"))
f$Perm <- str_sub(f$Perm,5)
f <- f%>%
  arrange(factor(Perm, levels = c("High","Medium","Low")))
f$Perm <- as.factor(f$Perm)
f$Perm <- ordered(f$Perm, levels = c("High","Medium","Low"))
f$City <- "Fai"
quantPerm <- rbind(e,f)

PermPlot <- ggplot(quantPerm, aes(fill=Perm, x=IncomeQuantile, y=n)) +
    geom_bar(width = 0.6, position="fill", stat="identity") +
    theme_classic() +
     scale_fill_viridis(discrete=T,option = "E") +
    theme(axis.text = element_text(size = 7))  +
    facet_wrap(~City, labeller = labeller(City = c("Anc" = "Anchorage","Fai" =
"Fairbanks"))) +
    theme_ipsum() +
    xlab("Income quartile")+
  ylab("% in hazard zone") +
  scale_y_continuous(labels = scales::percent) +
   theme(axis.title.y = element_text(vjust=+2)) +
  guides(fill=guide_legend(title="Assessed hazard level"))+
  theme(legend.position="bottom")
PermPlot
ggsave("Fig2-Perm.tif", plot = PermPlot, device = "tiff", path = NULL, scale = 1.0,
width = 140, height = 95, units = "mm",  dpi = 300, limitsize = T)

#write.csv(quantFire, "quantFire.csv")
#write.csv(quantIce, "quantIce.csv")
#write.csv(quantPerm, "quantPerm.csv")
```


## Table 2
Calculating number of multi-hazard affected households and proportions
```{r}
#estimated population proportions
ancAffected2 <- svytable(~FireAffectedPop+IceAffectedPop+PermAffectedPop,
design=w_ancData_trim)%>%
  round(digits=1)%>%
  as.data.frame()%>%
  mutate(total=sum(Freq),
         Prop=round(Freq/total,digits=2))
````

```
#write.csv(ancAffected2, "table2_anc.csv", row.names = F)
faiAffected2 <- svytable(~FireAffectedPop+IceAffectedPop+PermAffectedPop,
design=w_faiData_trim)%>%
  round(digits=1)%>%
  as.data.frame()%>%
  mutate(total=sum(Freq),
         Prop=round(Freq/total,digits=2))
#write.csv(faiAffected2, "table2_fai.csv", row.names = F)
#Calculating respondent count for this table

affCount <- with(data,
ifelse(FireAffectedPop==1|IceAffectedPop==1|PermAffectedPop==1,1,0 ))
sum(affCount)
```

```
#Table 3
```{r}
TableA2 <- rbind(
  svytable(~Q5, design=w_ancData_trim)%>%
  round(digits=1)%>%
  as.data.frame()%>%
  separate_rows(Q5, convert = T)%>%
  group_by(Q5)%>%
  summarise(Freq=sum(Freq))%>%
  ungroup()%>%
  mutate(Prop=round(Freq/ancParcels,digits=2)),
svytable(~Q5, design=w_faiData_trim)%>%
  round(digits=1)%>%
  as.data.frame()%>%
  separate_rows(Q5, convert = T)%>%
  group_by(Q5)%>%
  summarise(Freq=sum(Freq))%>%
  ungroup()%>%
  mutate(Prop=round(Freq/faiParcels,digits=2))
)

```

```{r}
Insurance <- rbind(
  svytable(~Q42n, design=w_ancData_trim)%>%
  round(digits=1)%>%
  as.data.frame()%>%
  separate_rows(Q42n, convert = T)%>%
  group_by(Q42n)%>%
  summarise(Freq=sum(Freq))%>%
  ungroup()%>%
  mutate(Prop=round(Freq/ancParcels,digits=2)),
svytable(~Q42n, design=w_faiData_trim)%>%
  round(digits=1)%>%
```
```

```
  as.data.frame()%>%
  separate_rows(Q42n, convert = T)%>%
  group_by(Q42n)%>%
  summarise(Freq=sum(Freq))%>%
  ungroup()%>%
  mutate(Prop=round(Freq/faiParcels,digits=2))
)
data$Q42n <- with(data,ifelse(is.na(Q42n),0,1))
Q42ncount <- sum(data$Q5n)
Q42ncount
```

##Table 4 Fire
City wide means, then mean subjective risk rating by hazard zone, mean fire mitigation cost over past 2 years, mean fire mitigation person hours per year on avg. over past 5 yrs, mean participation rate in Firewise, mean rating whether fire hazard has increased in past 10yr, all by hazard zone

```{r}
#Anchorage
svymean(~Q20_1, w_ancData_trim, na.rm=T) #Anchorage means
svymean(~FireMitCost, w_ancData_trim, na.rm=T)
svymean(~FireEffort, w_ancData_trim, na.rm=T)
svymean(~Firewise, w_ancData_trim, na.rm=T)
svymean(~FireTrend, w_ancData_trim, na.rm=T)
svymean(~PwrShutOff, w_ancData_trim, na.rm=T)
svymean(~FireAffectedPop, w_ancData_trim, na.rm=T)
svyby(~Q20_1,~Fire, w_ancData_trim, svymean, na.rm=T)
svyby(~FireAffectedPop,~Fire, w_ancData_trim, svymean, na.rm=T)
svyby(~FireMitCost,~Fire, w_ancData_trim, svymean, na.rm=T)
svyby(~FireEffort,~Fire, w_ancData_trim, svymean, na.rm=T)
svyby(~Firewise,~Fire, w_ancData_trim, svymean, na.rm=T)
svyby(~FireTrend,~Fire, w_ancData_trim, svymean, na.rm=T)
svyby(~PwrShutOff,~Fire, w_ancData_trim, svymean, na.rm=T)

#Fairbanks
svymean(~Q20_1, w_faiData_trim, na.rm=T) #Anchorage means
svymean(~FireMitCost, w_faiData_trim, na.rm=T)
svymean(~FireEffort, w_faiData_trim, na.rm=T)
svymean(~Firewise, w_faiData_trim, na.rm=T)
svymean(~FireTrend, w_faiData_trim, na.rm=T)
svymean(~PwrShutOff, w_faiData_trim, na.rm=T)
svymean(~FireAffectedPop, w_faiData_trim, na.rm=T)
svyby(~Q20_1,~Fire, w_faiData_trim, svymean, na.rm=T)
svyby(~FireAffectedPop,~Fire, w_faiData_trim, svymean, na.rm=T)
svyby(~FireMitCost,~Fire, w_faiData_trim, svymean, na.rm=T)
svyby(~FireEffort,~Fire, w_faiData_trim, svymean, na.rm=T)
svyby(~Firewise,~Fire, w_faiData_trim, svymean, na.rm=T)
svyby(~FireTrend,~Fire, w_faiData_trim, svymean, na.rm=T)
svyby(~PwrShutOff,~Fire, w_faiData_trim, svymean, na.rm=T)
```

````
#additional info on totals not presented in the table but in the text
svyratio(~Q46_2, ~FireMitCost, w_ancData_trim, na.rm=T) #Anchorage out of pocket ratio for fire mitigation costs
svyratio(~Q46_2, ~FireMitCost, w_faiData_trim, na.rm=T) #Fairbanks out of pocket ratio for fire mitigation costs

svytotal(~FireMitCost, w_ancData_trim, na.rm=T)
svytotal(~FireMitCost, w_faiData_trim, na.rm=T)

svytotal(~FireEffort, w_ancData_trim, na.rm=T)
svytotal(~FireEffort, w_faiData_trim, na.rm=T)

svytotal(~Firewise, w_ancData_trim, na.rm=T)
svytotal(~Firewise, w_faiData_trim, na.rm=T)
```

##Table 5  Ice
City wide means, then mean subjective risk rating by hazard zone, mean ice mitigation cost over past 2 years, mean damages and injury cost over past 2 years, proportion of households reporting damages or not that actually had damages, mean rating whether ice hazard has increased in past 10yr, all by hazard zone
```{r}
#Anchorage
svymean(~Q32_1, w_ancData_trim, na.rm=T) #means for Anchorage
svymean(~IceMitCost, w_ancData_trim, na.rm=T)
svymean(~InjTotCost, w_ancData_trim, na.rm=T)
svymean(~ice_injury, w_ancData_trim, na.rm=T)
svymean(~IceTrend, w_ancData_trim, na.rm=T)
svymean(~IceAffectedPop, w_ancData_trim, na.rm=T)
svyby(~Q32_1, ~Ice, w_ancData_trim, na.rm=T, svymean)
svyby(~IceMitCost, ~Ice, w_ancData_trim, na.rm=T, svymean)
svyby(~InjTotCost, ~Ice, w_ancData_trim, na.rm=T, svymean)
svyby(~ice_injury, ~Ice, w_ancData_trim, na.rm=T, svymean)
svyby(~IceTrend, ~Ice, w_ancData_trim, na.rm=T, svymean)
svyby(~IceAffectedPop,~Ice, w_ancData_trim, na.rm=T, svymean)

#Fairbanks
svymean(~Q32_1, w_faiData_trim, na.rm=T) #means for Fairbanks
svymean(~IceMitCost, w_faiData_trim, na.rm=T)
svymean(~InjTotCost, w_faiData_trim, na.rm=T)
svymean(~ice_injury, w_faiData_trim, na.rm=T)
svymean(~IceTrend, w_faiData_trim, na.rm=T)
svymean(~IceAffectedPop, w_faiData_trim, na.rm=T)
svyby(~Q32_1, ~Ice, w_faiData_trim, na.rm=T, svymean)
svyby(~IceMitCost, ~Ice, w_faiData_trim, na.rm=T, svymean)
svyby(~InjTotCost, ~Ice, w_faiData_trim, na.rm=T, svymean)
svyby(~ice_injury, ~Ice, w_faiData_trim, na.rm=T, svymean)
svyby(~IceTrend, ~Ice, w_faiData_trim, na.rm=T, svymean)
svyby(~IceAffectedPop,~Ice, w_faiData_trim, na.rm=T, svymean)
````

```
#additional info not presented in the table but in the text
svyratio(~Q27_2, ~IceMitCost, w_ancData_trim, na.rm=T) #out of pocket ratio for ice mitigation cost
svyratio(~Q27_2, ~IceMitCost, w_faiData_trim, na.rm=T) #out of pocket ratio for ice mitigation cost

svyratio(~Q25_2, ~InjTotCost, w_ancData_trim, na.rm=T) #out of pocket ratio for damages and health care cost
svyratio(~Q25_2, ~InjTotCost, w_faiData_trim, na.rm=T) #out of pocket ratio for damages and health care cost

svytotal(~IceMitCost, w_ancData_trim, na.rm=T)
svytotal(~IceMitCost, w_faiData_trim, na.rm=T)

svytotal(~InjTotCost, w_ancData_trim, na.rm=T)
svytotal(~InjTotCost, w_faiData_trim, na.rm=T)

svytotal(~ice_injury, w_ancData_trim, na.rm=T) #Number of households reporting injuries or damages from ice hazards
svytotal(~ice_injury, w_faiData_trim, na.rm=T)
```

##Table 6 Permafrost

City wide means, then mean subjective risk rating by hazard zone, mean permafrost mitigation cost since respondent lived at residence, proportion of households having reported sink holes and believing they are likely or somewhat likely related to permafrost, proportion of households believing permafrost hazard has increased in past 10yr, all by hazard zone

```{r}
#Anchorage
svymean(~Q41_1, w_ancData_trim, na.rm=T)
svymean(~PFMitCost, w_ancData_trim, na.rm=T)
svytotal(~PFMitCost, w_ancData_trim, na.rm=T)
svymean(~PermTrend, w_ancData_trim, na.rm=T)
svymean(~PermAffectedPop, w_ancData_trim, na.rm=T)
svyby(~Q41_1, ~Perm, w_ancData_trim, na.rm=T, svymean)
#svyby(~PFMitCost, ~Perm, w_ancData_trim, na.rm=T, svyquantile, quantiles=c(0.5,0.9))
svyby(~PFMitCost, ~Perm, w_ancData_trim, na.rm=T, svymean)
svyby(~PFMitCost, ~Perm, w_ancData_trim, na.rm=T, svytotal)
svyby(~PermTrend, ~Perm, w_ancData_trim, na.rm=T, svymean)
svyby(~PermAffectedPop, ~Perm, w_ancData_trim, na.rm=T, svymean)

#Fairbanks
svymean(~Q41_1, w_faiData_trim, na.rm=T)
svymean(~PFMitCost, w_faiData_trim, na.rm=T)
svytotal(~PFMitCost, w_faiData_trim, na.rm=T)
svymean(~PermTrend, w_faiData_trim, na.rm=T)
svymean(~PermAffectedPop, w_faiData_trim, na.rm=T)
svyby(~Q41_1, ~Perm, w_faiData_trim, na.rm=T, svymean)
```

````
#svyby(~PFMitCost, ~Perm, w_faiData_trim, na.rm=T, svyquantile,
quantiles=c(0.5,0.9))
svyby(~PFMitCost, ~Perm, w_faiData_trim, na.rm=T, svytotal)
svyby(~PFMitCost, ~Perm, w_faiData_trim, na.rm=T, svymean)
svyby(~PermTrend, ~Perm, w_faiData_trim, na.rm=T, svymean)
svyby(~PermAffectedPop, ~Perm, w_faiData_trim, na.rm=T, svymean)

#additional info not presented in the table but in the text
x<- svyratio(~Q48_2, ~PFMitCost, w_ancData_trim, na.rm=T) #out of pocket ratio for
permafrost mitigation cost
svytotal(~PFMitCost, w_ancData_trim, na.rm=T) #total permafrost mitigation
svymean(~PermInsure, w_ancData_trim, na.rm=T) #prop reported of hhs where insurance
pays perm damage
svyratio(~Q48_2, ~PFMitCost, w_faiData_trim, na.rm=T) #out of pocket ratio for
permafrost mitigation cost
svytotal(~PFMitCost, w_faiData_trim, na.rm=T) #total permafrost mitigation
svymean(~PermInsure, w_faiData_trim, na.rm=T) #prop reported of hhs where insurance
pays perm damage
```

### Sample size count for estimates presented in Tables 2, 6, and 8 from above
```{r}
ancNsizes <- ancData%>%

select(c("Parcel_ID","Q4","FireM","Fire","Ice","Perm","Q20_1","Q32_1","Q41_1","Fire
MitCost","IceMitCost","PFMitCost","FireTrend","IceTrend","PermTrend","Firewise","Fi
reEffort","PwrShutOff","Ice","InjTotCost","ice_injury","subsid"))
summary(ancNsizes)
faiNsizes <- faiData%>%

select(c("Parcel_ID","Q4","FireM","Fire","Ice","Perm","Q20_1","Q32_1","Q41_1","Fire
MitCost","IceMitCost","PFMitCost","FireTrend","IceTrend","PermTrend","Firewise","Fi
reEffort","PwrShutOff","Ice","InjTotCost","ice_injury","subsid"))
summary(faiNsizes)
```


##Other tables and figures
We created weighted proportion tables (WPT), by following
https://zacharylhertz.github.io/posts/2021/06/survey-package
Steps: create separate dataframe of all the variables that need WPT, then separate
comma-delimited values and turn into long format

###Figure 3, first part
first part, wildfire mitigation activity on house of residence
```{r}
Q11labels <- c("Installed fire resistant siding","Installed fire resistant
roofing","Installed gutter guards","Installed screening over vents","Installed a
chimney spark arrester","Cleaned roof and gutters","Closed eaves with horizontal
soffit","Other","None","No response")
````

````
#Table 3
Table3 <- rbind(
  svytable(~Q11, design=w_ancData_trim)%>%
  round(digits=1)%>%
  as.data.frame()%>%
  separate_rows(Q11, convert = T)%>%
  group_by(Q11)%>%
  summarise(Freq=sum(Freq))%>%
  ungroup()%>%
  mutate(Prop=round(Freq/ancParcels,digits=2),
         City="Anchorage",
         Label=Q11labels),
faiQ11 <- svytable(~Q11, design=w_faiData_trim)%>%
  round(digits=1)%>%
  as.data.frame()%>%
  separate_rows(Q11, convert = T)%>%
  group_by(Q11)%>%
  summarise(Freq=sum(Freq))%>%
  ungroup()%>%
  mutate(Prop=round(Freq/faiParcels,digits=2),
         City="Fairbanks",
         Label=Q11labels)
)

#reorder percentages
Fig3Data <- Table3%>%
  arrange(City,Prop)%>%
  mutate(id=seq(1:20))%>%
  mutate(Percentage=Prop*100, .keep="all")

Fig3a <- ggplot(Fig3Data, aes(fill=City, y=Percentage, x=reorder(Label,id))) +
    geom_bar(position="dodge", stat="identity") +
    theme_classic() +
    theme(axis.text.x = element_text(vjust = 0.5, hjust=0.5))+
    labs(x="",y="") + theme(legend.title = element_blank() , legend.position
=c(0.85,0.25))+
    scale_y_continuous(breaks = seq(5, 65, by = 5),limits=c(0,65))  +
  scale_fill_viridis(discrete=T,option = "E") +
  theme(axis.text = element_text(size = 7))  +
  theme(legend.position="none")+
  coord_flip()
Fig3a
```

###Figure 3, second part
second part, wildfire mitigation activity on land parcel
```{r}
Q12labels <- c("Removed all conifers within 15ft of house","Thinned conifers
between 15 and 100ft from the house","Removed all dry and dead vegetation","Removed
shrubs beneath trees","Pruned limbs of mature conifers","Thinned black
````

```
spruce","Kept lawn at less than 3in and watered","Kept trees healthy and
watered","Kept areas under stairs and decks free of debris","Installed a 3ft
perimeter around home with non-burnables","Provided 15ft of clearance between
chimney and trees","Other","None","Removed dead trees","Moved firewood at least
30ft away from house","Kept external fire sprinkler system on property","Planted
fire-resistant vegetation such as larch","No response")
#Table 4
Table4 <- rbind(
  svytable(~Q12, design=w_ancData_trim)%>%
  round(digits=1)%>%
  as.data.frame()%>%
  separate_rows(Q12, convert = T)%>%
  group_by(Q12)%>%
  summarise(Freq=sum(Freq))%>%
  ungroup()%>%
  mutate(Prop=round(Freq/ancParcels,digits=2),
         City="Anchorage",
         Label=Q12labels),
svytable(~Q12, design=w_faiData_trim)%>%
  round(digits=1)%>%
  as.data.frame()%>%
  separate_rows(Q12, convert = T)%>%
  group_by(Q12)%>%
  summarise(Freq=sum(Freq))%>%
  ungroup()%>%
  mutate(Prop=round(Freq/faiParcels,digits=2),
         City="Fairbanks",
         Label=Q12labels)
)

#reorder percentages
Fig4Data <- Table4%>%
  arrange(City,Prop)%>%
  mutate(id=seq(1:36))%>%
  mutate(Percentage=Prop*100, .keep="all")

Fig3b <- ggplot(Fig4Data, aes(fill=City, y=Percentage, x=reorder(Label,id))) +
    geom_bar(position="dodge", stat="identity") +
    theme_classic() +
    #theme(axis.text.x = element_text(vjust = 0.5, hjust=1))+
    labs(x="",y="percent") + theme(legend.title = element_blank() , legend.position
=c(0.85,0.25))+
    scale_y_continuous(breaks = seq(5, 65, by = 5), limits=c(0,65) )  +
  scale_fill_viridis(discrete=T,option = "E") +
  theme(axis.text = element_text(size = 7))  +
  coord_flip()
Fig3b

#Combining Fig 3 and 4
theme_set(theme_cowplot(font_size=7))
```

````
Fig3 <- plot_grid(Fig3a, Fig3b, labels = c("House", "Parcel"), ncol = 1, align =
"v", label_x=0, label_y=1, hjust=0, label_size=11, rel_heights = c(1,1.7))
Fig3
#save plot
#ggsave("Fig3.tif", plot = Fig3, device = "tiff", path = NULL, scale = 1, width =
140, height = 180, units = "mm", dpi = 300, limitsize = T)
```

###Figure 4
How households are affected by ice hazards
```{r}
Q24labels <- c("Car or other accident","Lost or damaged property","Injury","Kids
missed school or day-care","Other impact","Missed work","Left work early or arrived
late","School or day-care closures","Unable to participate in planned
activities","No impact","Service outage","No response")

#Table 5
Table5 <- rbind(
  svytable(~Q24, design=w_ancData_trim)%>%
  round(digits=1)%>%
  as.data.frame()%>%
  separate_rows(Q24, convert = T)%>%
  group_by(Q24)%>%
  summarise(Freq=sum(Freq))%>%
  ungroup()%>%
  mutate(Prop=round(Freq/ancParcels,digits=2),
         City="Anchorage",
         Label=Q24labels),
svytable(~Q24, design=w_faiData_trim)%>%
  round(digits=1)%>%
  as.data.frame()%>%
  separate_rows(Q24, convert = T)%>%
  group_by(Q24)%>%
  summarise(Freq=sum(Freq))%>%
  ungroup()%>%
 mutate(Prop=round(Freq/faiParcels,digits=2),
        City="Fairbanks",
         Label=Q24labels)
)

#reorder percentages
Fig5Data <- Table5%>%
  arrange(City,Prop)%>%
  mutate(id=seq(1:24))%>%
  mutate(Percentage=Prop*100, .keep="all")

Fig5 <- ggplot(Fig5Data, aes(fill=City, y=Percentage, x=reorder(Label,id))) +
    geom_bar(width=0.6, position="dodge", stat="identity") +
    theme_classic() +
    #theme(axis.text.x = element_text(vjust = 0.5, hjust=1))+
````

````
    labs(x="",y="percent") + theme(legend.title = element_blank() , legend.position =c(0.85,0.25))+
    scale_y_continuous(breaks = seq(5, 80, by = 5))  +
  scale_fill_viridis(discrete=T,option = "E") +
  theme(axis.text = element_text(size = 10))  +
  coord_flip()
Fig5
#ggsave("Fig4.tif", plot = Fig5, device = "tiff", path = NULL, scale = 1, width = 174, height = 80, units = "mm", dpi = 300, limitsize = TRUE)
```

###Figure 5
Impacts of permafrost thaw
```{r}
Q38labels <- c("Utility access","Road access","Other impact","No impact","Decreased property value","Decreased ability to sell property","Changed vegetation","Made property unsafe","Septic tank","Foundation","Lawn and landscaping","Ground surface collapse","Pools of standing water and erosion gullies","No response")

#Table 7
Table7 <- rbind(
  svytable(~Q38, design=w_ancData_trim)%>%
  round(digits=1)%>%
  as.data.frame()%>%
  separate_rows(Q38, convert = T)%>%
  group_by(Q38)%>%
  summarise(Freq=sum(Freq))%>%
  ungroup()%>%
  mutate(Prop=round(Freq/ancParcels,digits=2),
         City="Anchorage",
         Label=Q38labels)%>%
    na.omit(Q38),
svytable(~Q38, design=w_faiData_trim)%>%
  round(digits=1)%>%
  as.data.frame()%>%
  separate_rows(Q38, convert = T)%>%
  group_by(Q38)%>%
  summarise(Freq=sum(Freq))%>%
  ungroup()%>%
  mutate(Prop=round(Freq/faiParcels,digits=2),
         City="Fairbanks",
         Label=Q38labels)%>%
  na.omit(Q38)
)
#reorder percentages
Fig6Data <- Table7%>%
  arrange(City,Prop)%>%
  mutate(id=seq(1:26))%>%
  mutate(Percentage=Prop*100, .keep="all")
````

~~~
Fig6 <- ggplot(Fig6Data, aes(fill=City, y=Percentage, x=reorder(Label,id))) +
    geom_bar(width = 0.6, position="dodge", stat="identity") +
    theme_classic() +
    #theme(axis.text.x = element_text(vjust = 0.5, hjust=1))+
    labs(x="",y="percent") + theme(legend.title = element_blank() , legend.position
=c(0.85,0.25))+
    scale_y_continuous(breaks = seq(5, 80, by = 5))  +
  scale_fill_viridis(discrete=T,option = "E") +
  theme(axis.text = element_text(size = 10))  +
  coord_flip()
Fig6
#ggsave("Fig5.tif", plot = Fig6, device = "tiff", path = NULL, scale = 1, width =
174, height = 80, units = "mm", dpi = 300, limitsize = TRUE)
```

###Figure 6
Permafrost mitigation actions
```{r}
Q39labels <- c("Built elevated structure on piling or post and pad","Planted shrubs
for shading around building perimeter","Dug out frozen soil and backfilled with
gravel","Installed gutters to route water away from foundation","Installed above
ground septic system","Built up a gravel pad","Built extended eaves","Built in an
adjustable foundation","Installed active cooling system","Other","None","No
response")

#Table 9
ancPF <- svytable(~Q39, design=w_ancData_trim)%>%
  round(digits=1)%>%
  as.data.frame()%>%
  separate_rows(Q39, convert = T)%>%
  group_by(Q39)%>%
  summarise(Freq=sum(Freq))%>%
  ungroup()%>%
  mutate(Prop=round(Freq/ancParcels,digits=2))
Q39 <- c(5,9)
Freq <- c(0,0)
Prop <- c(0,0)
ancPF2 <- rbind(data.frame(Q39,Freq,Prop),ancPF)
ancPF3 <- ancPF2[order(ancPF2$Q39),]

Table9 <- rbind(
  ancPF3%>%
  mutate(City="Anchorage",
         Label=Q39labels)%>%
    na.omit(Q39),
svytable(~Q39, design=w_faiData_trim)%>%
  round(digits=1)%>%
  as.data.frame()%>%
  separate_rows(Q39, convert = T)%>%
  group_by(Q39)%>%
~~~

````
  summarise(Freq=sum(Freq))%>%
  ungroup()%>%
  mutate(Prop=round(Freq/faiParcels,digits=2),
         City="Fairbanks",
         Label=Q39labels)%>%
  na.omit(Q39)
)

#reorder percentages
Fig7Data <- Table9%>%
  arrange(City,Prop)%>%
  mutate(id=seq(1:22))%>%
  mutate(Percentage=Prop*100, .keep="all")

Fig7 <- ggplot(Fig7Data, aes(fill=City, y=Percentage, x=reorder(Label,id))) +
    geom_bar(width = 0.6, position="dodge", stat="identity") +
    theme_classic() +
    #theme(axis.text.x = element_text(vjust = 0.5, hjust=1))+
    labs(x="",y="percent") + theme(legend.title = element_blank() , legend.position
=c(0.85,0.13))+
    scale_y_continuous(breaks = seq(5, 100, by = 5))  +
  scale_fill_viridis(discrete=T,option = "E") +
  theme(axis.text = element_text(size = 10))  +
  coord_flip()
Fig7
#ggsave("Fig6.tif", plot = Fig7, device = "tiff", path = NULL, scale = 1, width =
174, height = 100, units = "mm", dpi = 300, limitsize = TRUE)
```

###Information presented in the paper's text but not as tables
```{r}
#Internet service interruption length
ancQ6_1 <- svytable(~Q6_1, design=w_ancData_trim)%>%
  round(digits=1)%>%
  as.data.frame()%>%
  separate_rows(Q6_1, convert = T)%>%
  group_by(Q6_1)%>%
  na.omit()%>%
  summarise(Freq=sum(Freq))%>%
  ungroup()%>%
  mutate(Prop=round(Freq/ancParcels,digits=2))
faiQ6_1 <- svytable(~Q6_1, design=w_faiData_trim)%>%
  round(digits=1)%>%
  as.data.frame()%>%
  separate_rows(Q6_1, convert = T)%>%
  group_by(Q6_1)%>%
  na.omit()%>%
  summarise(Freq=sum(Freq))%>%
  ungroup()%>%
  mutate(Prop=round(Freq/faiParcels,digits=2))
````

```
#Internet service interruption length
ancQ6_2 <- svytable(~Q6_2, design=w_ancData_trim)%>%
  round(digits=1)%>%
  as.data.frame()%>%
  separate_rows(Q6_2, convert = T)%>%
  group_by(Q6_2)%>%
  na.omit()%>%
  summarise(Freq=sum(Freq))%>%
  ungroup()%>%
  mutate(Prop=round(Freq/ancParcels,digits=2))
faiQ6_2 <- svytable(~Q6_2, design=w_faiData_trim)%>%
  round(digits=1)%>%
  as.data.frame()%>%
  separate_rows(Q6_2, convert = T)%>%
  group_by(Q6_2)%>%
  na.omit()%>%
  summarise(Freq=sum(Freq))%>%
  ungroup()%>%
  mutate(Prop=round(Freq/faiParcels,digits=2))
#Electric Service interruptions due to ice hazards
ancQ29_1 <- svytable(~Q29_1, design=w_ancData_trim)%>%
  round(digits=1)%>%
  as.data.frame()%>%
  separate_rows(Q29_1, convert = T)%>%
  group_by(Q29_1)%>%
  na.omit()%>%
  summarise(Freq=sum(Freq))%>%
  ungroup()%>%
  mutate(Prop=round(Freq/ancParcels,digits=2))
faiQ29_1 <- svytable(~Q29_1, design=w_faiData_trim)%>%
  round(digits=1)%>%
  as.data.frame()%>%
  separate_rows(Q29_1, convert = T)%>%
  group_by(Q29_1)%>%
  na.omit()%>%
  summarise(Freq=sum(Freq))%>%
  ungroup()%>%
  mutate(Prop=round(Freq/faiParcels,digits=2))
#Internet Service interruptions due to ice hazards
ancQ29_2 <- svytable(~Q29_2, design=w_ancData_trim)%>%
  round(digits=1)%>%
  as.data.frame()%>%
  separate_rows(Q29_2, convert = T)%>%
  group_by(Q29_2)%>%
  na.omit()%>%
  summarise(Freq=sum(Freq))%>%
  ungroup()%>%
  mutate(Prop=round(Freq/ancParcels,digits=2))
faiQ29_2 <- svytable(~Q29_2, design=w_faiData_trim)%>%
  round(digits=1)%>%
```

```
  as.data.frame()%>%
  separate_rows(Q29_2, convert = T)%>%
  group_by(Q29_2)%>%
  na.omit()%>%
  summarise(Freq=sum(Freq))%>%
  ungroup()%>%
mutate(Prop=round(Freq/ancParcels,digits=2))
#sink holes
ancQ35<- svytable(~Q35, design=w_ancData_trim)%>%
  round(digits=1)%>%
  as.data.frame()%>%
  separate_rows(Q35, convert = T)%>%
  group_by(Q35)%>%
  summarise(Freq=sum(Freq))%>%
  ungroup()%>%
  mutate(Prop=round(Freq/ancParcels,digits=2))
faiQ35 <- svytable(~Q35, design=w_faiData_trim)%>%
  round(digits=1)%>%
  as.data.frame()%>%
  separate_rows(Q35, convert = T)%>%
  group_by(Q35)%>%
  summarise(Freq=sum(Freq))%>%
  ungroup()%>%
  mutate(Prop=round(Freq/faiParcels,digits=2))
#concern about permafrost
ancQ37<- svytable(~Q37, design=w_ancData_trim)%>%
  round(digits=1)%>%
  as.data.frame()%>%
  separate_rows(Q37, convert = T)%>%
  group_by(Q37)%>%
  summarise(Freq=sum(Freq))%>%
  ungroup()%>%
  mutate(Prop=round(Freq/ancParcels,digits=2))
faiQ37 <- svytable(~Q37, design=w_faiData_trim)%>%
  round(digits=1)%>%
  as.data.frame()%>%
  separate_rows(Q37, convert = T)%>%
  group_by(Q37)%>%
  summarise(Freq=sum(Freq))%>%
  ungroup()%>%
  mutate(Prop=round(Freq/faiParcels,digits=2))
#reasons for why homeowners have no insurance covering wildfire
ancQ43<- svytable(~Q43, design=w_ancData_trim)%>%
  round(digits=1)%>%
  as.data.frame()%>%
  separate_rows(Q43, convert = T)%>%
  group_by(Q43)%>%
  summarise(Freq=sum(Freq))%>%
  ungroup()%>%
  mutate(Prop=round(Freq/ancParcels,digits=2))
```

````
faiQ43<- svytable(~Q43, design=w_faiData_trim)%>%
  round(digits=1)%>%
  as.data.frame()%>%
  separate_rows(Q43, convert = T)%>%
  group_by(Q43)%>%
  summarise(Freq=sum(Freq))%>%
  ungroup()%>%
  mutate(Prop=round(Freq/faiParcels,digits=2))

#write.csv(ancQ43,"ancQ43.csv", row.names = F)
#insurance companies providing
ancQ45 <- svytable(~Q45, design=w_ancData_trim)%>%
  round(digits=1)%>%
  as.data.frame()%>%
  separate_rows(Q45, convert = T)%>%
  group_by(Q45)%>%
  summarise(Freq=sum(Freq))%>%
  ungroup()%>%
  mutate(Prop=round(Freq/faiParcels,digits=2))
#check code here!
faiQ45 <- svytable(~Q45, design=w_faiData_trim)%>%
  round(digits=1)%>%
  as.data.frame()%>%
  separate_rows(Q45, convert = T)%>%
  group_by(Q45)%>%
  summarise(Freq=sum(Freq))%>%
  ungroup()%>%
  mutate(total=sum(Freq),
         Prop=round(Freq/total,digits=2))
#insurance company requiring home to be prepared for wildfire
faiQ45 <- svytable(~Q45+Q44, design=w_ancData_trim)%>%
  round(digits=1)%>%
  as.data.frame()
```

###Tables in Online Resource 3
```{r}

#Table A2
TableA3 <- rbind(
  svytable(~Q15, design=w_ancData_trim)%>%
  round(digits=1)%>%
  as.data.frame()%>%
  separate_rows(Q15, convert = T)%>%
  group_by(Q15)%>%
  summarise(Freq=sum(Freq))%>%
  ungroup()%>%
  mutate(total=sum(Freq),
         Prop=round(Freq/total,digits=2)),
svytable(~Q15, design=w_faiData_trim)%>%
````

```
  round(digits=1)%>%
  as.data.frame()%>%
  separate_rows(Q15, convert = T)%>%
  group_by(Q15)%>%
  summarise(Freq=sum(Freq))%>%
  ungroup()%>%
  mutate(total=sum(Freq),
         Prop=round(Freq/total,digits=2))
)
#Table A3, Gov effectiveness and preparedness for fire
TableA4Eff<- rbind(
  svytable(~Q8, design=w_ancData_trim)%>%
  round(digits=1)%>%
  as.data.frame()%>%
  separate_rows(Q8, convert = T)%>%
  group_by(Q8)%>%
  summarise(Freq=sum(Freq))%>%
  ungroup()%>%
  mutate(total=sum(Freq),
        Prop=round(Freq/total,digits=2)),
svytable(~Q8, design=w_faiData_trim)%>%
  round(digits=1)%>%
  as.data.frame()%>%
  separate_rows(Q8, convert = T)%>%
  group_by(Q8)%>%
  summarise(Freq=sum(Freq))%>%
  ungroup()%>%
  mutate(total=sum(Freq),
         Prop=round(Freq/total,digits=2))
)

TableA4Prep <- rbind(
  svytable(~Q9, design=w_ancData_trim)%>%
  round(digits=1)%>%
  as.data.frame()%>%
  separate_rows(Q9, convert = T)%>%
  group_by(Q9)%>%
  summarise(Freq=sum(Freq))%>%
  ungroup()%>%
  mutate(total=sum(Freq),
         Prop=round(Freq/total,digits=2)),
svytable(~Q9, design=w_faiData_trim)%>%
  round(digits=1)%>%
  as.data.frame()%>%
  separate_rows(Q9, convert = T)%>%
  group_by(Q9)%>%
  summarise(Freq=sum(Freq))%>%
  ungroup()%>%
  mutate(total=sum(Freq),
         Prop=round(Freq/total,digits=2))
```

```
)
#Table A4, responsibilities of private landowners
TableA5 <- rbind(
  svytable(~Q10, design=w_ancData_trim)%>%
  round(digits=1)%>%
  as.data.frame()%>%
  separate_rows(Q10, convert = T)%>%
  group_by(Q10)%>%
  summarise(Freq=sum(Freq))%>%
  ungroup()%>%
  mutate(Prop=round(Freq/ancParcels,digits=2)),
svytable(~Q10, design=w_faiData_trim)%>%
  round(digits=1)%>%
  as.data.frame()%>%
  separate_rows(Q10, convert = T)%>%
  group_by(Q10)%>%
  summarise(Freq=sum(Freq))%>%
  ungroup()%>%
  mutate(Prop=round(Freq/faiParcels,digits=2))
)
#Table A5
TableA6 <- rbind(
  svytable(~Q14, design=w_ancData_trim)%>%
  round(digits=1)%>%
  as.data.frame()%>%
  separate_rows(Q14, convert = T)%>%
  group_by(Q14)%>%
  summarise(Freq=sum(Freq))%>%
  ungroup()%>%
  mutate(Prop=round(Freq/ancParcels,digits=2)),
svytable(~Q14, design=w_faiData_trim)%>%
  round(digits=1)%>%
  as.data.frame()%>%
  separate_rows(Q14, convert = T)%>%
  group_by(Q14)%>%
  summarise(Freq=sum(Freq))%>%
  ungroup()%>%
mutate(Prop=round(Freq/faiParcels,digits=2))
)
#Table A6
TableA7 <- rbind(
  svytable(~Q26, design=w_ancData_trim)%>%
  round(digits=1)%>%
  as.data.frame()%>%
  separate_rows(Q26, convert = T)%>%
  group_by(Q26)%>%
  summarise(Freq=sum(Freq))%>%
  ungroup()%>%
  mutate(Prop=round(Freq/ancParcels,digits=2)),
svytable(~Q26, design=w_faiData_trim)%>%
```

~~~
  round(digits=1)%>%
  as.data.frame()%>%
  separate_rows(Q26, convert = T)%>%
  group_by(Q26)%>%
  summarise(Freq=sum(Freq))%>%
  ungroup()%>%
  mutate(Prop=round(Freq/ancParcels,digits=2))
)
#Table A7
ancQ28<- svytable(~Q28, design=w_ancData_trim)%>%
  round(digits=1)%>%
  as.data.frame()%>%
  separate_rows(Q28, convert = T)%>%
  group_by(Q28)%>%
  summarise(Freq=sum(Freq))%>%
  ungroup()%>%
  mutate(total=sum(Freq),
         Prop=round(Freq/total,digits=2))
faiQ28 <- svytable(~Q28, design=w_faiData_trim)%>%
  round(digits=1)%>%
  as.data.frame()%>%
  separate_rows(Q28, convert = T)%>%
  group_by(Q28)%>%
  summarise(Freq=sum(Freq))%>%
  ungroup()%>%
  mutate(total=sum(Freq),
         Prop=round(Freq/total,digits=2))
```
~~~
